# Analysis of Genomic Characteristics and Transmission Routes of Patients With Confirmed SARS-CoV-2 in Southern California During the Early Stage of the US COVID-19 Pandemic

**DOI:** 10.1001/jamanetworkopen.2020.24191

**Published:** 2020-10-07

**Authors:** Wenjuan Zhang, John Paul Govindavari, Brian D. Davis, Stephanie S. Chen, Jong Taek Kim, Jianbo Song, Jean Lopategui, Jasmine T. Plummer, Eric Vail

**Affiliations:** 1 Molecular Pathology Laboratory, Department of Pathology and Laboratory Medicine, Cedars-Sinai Medical Center, Los Angeles, California; 2 Center for Bioinformatics and Functional Genomics, Department of Biomedical Sciences, Cedars-Sinai Medical Center, Los Angeles, California; 3 Applied Genomics, Computation and Translational Core, Cedars-Sinai Cancer Center, Los Angeles, California

## Abstract

**Importance:**

In late December 2019, an outbreak of a novel severe acute respiratory syndrome coronavirus 2 (SARS-CoV-2) emerged in Wuhan, China. Data on the routes of transmission to Los Angeles, California, the US West Coast epicenter for coronavirus disease 2019 (COVID-19), and subsequent community spread are limited.

**Objective:**

To determine the transmission routes of SARS-CoV-2 to Southern California and elucidate local community spread within the Los Angeles metropolitan area.

**Design, Setting, and Participants:**

This case series included 192 consecutive patients with reverse transcription–polymerase chain reaction (RT-PCR) test results positive for SARS-CoV-2 who were evaluated at Cedars-Sinai Medical Center in Los Angeles, California, from March 22 to April 15, 2020. Data analysis was performed from April to May 2020.

**Main Outcomes and Measures:**

SARS-CoV-2 viral genomes were sequenced. Los Angeles isolates were compared with genomes from global subsampling and from New York, New York; Washington state; and China to determine potential sources of viral dissemination. Demographic data and outcomes were collected.

**Results:**

The cohort included 192 patients (median [interquartile range] age, 59.5 [43-75] years; 110 [57.3%] men). The genetic characterization of SARS-CoV-2 isolates in the Los Angeles population pinpointed community transmission of 13 patients within a 3.81 km^2^ radius. Variation landscapes of this case series also revealed a cluster of 10 patients that contained 5 residents at a skilled nursing facility, 1 resident of a nearby skilled nursing facility, 3 health care workers, and a family member of a resident of one of the skilled nursing facilities. Person-to-person transmission was detected in a cluster of 5 patients who shared the same single-nucleotide variation in their SARS-CoV-2 genomes. High viral genomic diversity was identified: 20 Los Angeles isolates (15.0%) resembled SARS-CoV-2 genomes from Asia, while 109 Los Angeles isolates (82.0%) were similar to isolates originating from Europe. Analysis of other common respiratory viral pathogens did not reveal coinfection in the cohort.

**Conclusions and Relevance:**

These findings highlight the precision of detecting person-to-person transmission and accurate contact tracing directly through SARS-CoV-2 genome isolation and sequencing. Development and application of phylogenetic analyses from the Los Angeles population established connections between COVID-19 clusters locally and throughout the US.

## Key Points

**Question:**

During the early phase of the outbreak, what were the transmission routes and genomic characteristics of severe acute respiratory syndrome coronavirus 2 (SARS-CoV-2) spread in Los Angeles, California?

**Findings:**

This case series of 192 patients found that 82% of SARS-CoV-2 isolates from Los Angeles shared closest similarity to those originating in Europe vs those from Asia (15%). Using the variation signature of the viral genomes, 2 main clusters were identified, with the top variants sharing genomic features from European SARS-CoV-2 isolates, and several subclusters of SARS-CoV-2 outbreaks represented trackable community spread in Los Angeles.

**Meaning:**

These findings suggest that SARS-CoV-2 genomes in Los Angeles were predominantly related to the isolates originating from Europe, which are similar to viral strain distributions in New York, New York; a smaller subgroup of SARS-CoV-2 genomes shared similarities to those from originating from Asia, indicating multiple sources of viral introduction within the Los Angeles community.

## Teaser

This case series examines the genomic characteristics of severe acute respiratory syndrome coronavirus 2 (SARS-CoV-2) infections and transmission routes during the early stages of the coronavirus disease 2019 (COVID-19) outbreak in Los Angeles, California.

## Introduction

The emergence of the coronavirus disease 2019 (COVID-19) global pandemic caused by severe acute respiratory syndrome coronavirus 2 (SARS-CoV-2)^1^ presents the scientific community with an urgent need to understand all aspects of this novel virus. The SARS-CoV-2 genome sequences deposited in public databases^2,3^ are pivotal resources in understanding its virulence and for guiding approaches to therapeutics and vaccines.^4^ Assessing core genomic features across all global populations can be used for comparative analysis to identify features unique to SARS-CoV-2 as well as assist in epidemiologic and public health endeavors.^2,5,6,7,8,9,10,11,12,13,14,15^

SARS-CoV-2 is a coronavirus with a 29 903–base pair (bp) single-stranded RNA genome^16^ containing 14 open reading frames and 27 estimated proteins.^17^ Viral genome annotation can assess the conserved wild-type sequence across all patients with COVID-19. Genomic epidemiology has emerged as a useful tool to track sources of transmission and SARS-CoV-2 evolution within communities and throughout the world.^9,10,13,18^ The consortium Global Initiative on Sharing All Influenza Data (GISAID)^2,3^ classifies the global distribution of SARS-CoV-2 into 2 main clades differing in their origins: (1) clade 19A, originating from China, and (2) clade 20A, originating form Europe. Clade 20B was seeded by a strain from China, but once in Europe, its variation profile became the predominant strain of the European pandemic.^19^

The first patient with confirmed COVID-19 in the US presented on January 19, 2020, in Washington state.^20^ While Seattle recorded the first observed transmission of SARS-CoV-2 from China, the largest SARS-CoV-2 outbreak in the US to date was in New York, New York.^9,12^ New York isolates were seeded on multiple introductions from Europe.^9^ A study by Deng et al^13^ reported that the early transmission of SARS-CoV-2 in the US West Coast originated mainly from China and Washington state (31 of 36 patients), with only 5 patients found to have SARS-CoV-2 infection sharing lineage with the New York outbreak. The genomic epidemiology of SARS-CoV-2 supports the current belief that isolates from China have primarily seeded the original COVID-19 outbreak on the US West Coast and the European isolates seeded the pandemic in New York (and the US East Coast).

Los Angeles, California, is the largest city on the US West Coast and had its first patient with confirmed COVID-19 in late January 2020.^21^ Accordingly, it was one of the first major US cities to take precautionary measures and restrict the population to their homes as fatalities increased in early March 2020.^22^ As of August 10, 2020, more than 200 000 confirmed SARS-COV-2–positive cases and 4996 COVID-19–related^3^ deaths have been recorded in Los Angeles county. Cedars-Sinai Medical Center (CSMC), located in Los Angeles, serves more than 1 million people and is the largest health service center west of the Mississippi River. A reverse transcription–polymerase chain reaction (RT-PCR) diagnostic test for SARS-CoV-2 infection was adopted March 21, 2020, allowing our clinical laboratory to rapidly screen and identify patients with SARS-CoV-2 infection. After transmission from China, our timeline for SARS-CoV-2 infection follows other reported introductions into different global populations.^5,11,14,15,23,24,25,26^ At the time of our study, the only Los Angeles SARS-CoV-2 genome deposited in GISAID was not linked to a particular model of introduction.^3^ Based on these cumulative findings, we hypothesize the local Los Angeles community was likely exposed to a US West Coast SARS-CoV-2 strain, which was directly transmitted from China. In an effort to further understand this evolving virus, we sought to perform next-generation sequencing (NGS) analysis on patients with confirmed SARS-CoV-2 infection. We conducted phylogenetic analyses on this unique West Coast population to identify local community spread within the greater Los Angeles area. A broad geographic distribution comparison of SARS-CoV-2 isolates in Southern California from early in the COVID-19 US outbreak with isolates in New York, Washington state, and China was conducted to ascertain transmission pathways of SARS-CoV-2 dissemination into Los Angeles. In this case series, we report potential sources of SARS-CoV-2 introduction into the Los Angeles community.

## Methods

### Sample Collection

Appropriate regulatory review was completed by the CSMC Office of Research Compliance and Quality Improvement. A waiver of informed consent was granted per institutional policy because the study did not require interaction or intervention with participants, posed no more than minimal risk to privacy of individuals, did not impact patients’ clinical care, could not be practically conducted without access to protected health information, and a requirement to obtain consent would render the research impracticable, as some patients were no longer receiving care at time of the study. Clinical specimens were collected by nasopharyngeal swabs from patients with COVID-19–like symptoms from March 22 to April 15, 2020. Associated clinical and demographic data were extracted from the electronic medical record. This study followed the Strengthening the Reporting of Observational Studies in Epidemiology (STROBE) reporting guideline for cohort studies.

### Sample Preparation

Total nucleic acid was extracted using the QIAamp Viral RNA Mini Kit on the QIAcube Connect (Qiagen). All patients were first assessed by RT-PCR (Accelerate Technologies) for SARS-CoV-2 viral RNA. The nucleic acid was screened for the presence of SARS-CoV-2 using real-time single-plex RT-PCR for the SARS-CoV-2 *nsp3* gene. All samples were diagnostically SARS-CoV-2–positive with amplification of the targeted region crossing the threshold before 40 cycles. In total, 192 SARS-CoV-2–positive samples were used for parallel NGS analysis.

### Targeted NGS and Phylogenetic Analyses

All samples were quantified by Qubit, and 100 ng of total RNA were processed for first strand and second strand complementary DNA synthesis using NEBNext Ultra II Directional RNA Library Prep Kit modular workflow (New England Biolabs) according to the manufacturers’ recommendations. Target enrichment of 200 ng of complementary DNA was performed using the Nextera Flex library preparation kit combined with the Illumina viral respiratory panel and DNA unique dual indices (Illumina). After enrichment, all samples were pooled, loaded, and sequenced on a NovaSeq Illumina platform (150 bp paired-end). Sequencing reads were mapped to 41 respiratory virus genomes, including the SARS-CoV-2 reference genome (NCI_045512.2) (eTable 1 in the Supplement) with BWA-MEM software version 0.7.17-r1188.^27^ All samples with greater than 50% of the SARS-CoV-2 genome covered with more than 10× depth were included in the study, which totaled 133 isolates. These genomes passed quality control assessment by Nextclade^28^ and were retained for downstream phylogenetic analysis. Duplicated reads were labeled with Picard,^29^ and BCFtools^30^ was used to generate consensus sequences. Data used in this study have been deposited to GISAID (eTable 2 in the Supplement). The mapping ratio was calculated by Samtools,^31^ and the Pearson correlation coefficient was calculated between mapping ratio and threshold cycle (Ct) value obtained by RT-PCR with R statistical software version 3.6.3 (R Project for Statistical Computing).

 Samples from Washington state, New York, and China were downloaded from the GISAID EpiCoV database as of May 18, 2020,^2^ and only complete sequences were included, totaling 3398 SARS-CoV-2 genomes (eTable 3 in the Supplement).

### Statistical Analysis

Multiple sample alignment was performed with MAFFT version 7.464^32^ and a maximum likelihood tree reconstruction was performed with IQ-TREE version 2.0.3^33^ with the best-fit model chosen based on bayesian information criterion. Branch support was inferred using 1000 bootstrap replicates. Maximum-likelihood phylodynamic analysis was inferred by collection date with TreeTime^34^ using generalized time reversible model. Tree visualizations were performed with FigTree version 1.4.4^35^ and iTOL version 5.6.1.^36^ Subsampling with global background was performed by NextClade with CSMC samples. Sample percentages were calculated based on their distribution within Nextstrain global clades.^3^ As of September 2020, global SARS-CoV-2 clades were designated into clades 19A and 19B of Asian origin and clades 20A, 20B, and 20C of European origin. *P* values were 2-sided, and statistical significance was set at .05.

## Results

### Sequenced SARS-CoV-2 Specimens From CSMC

**Figure 1.**
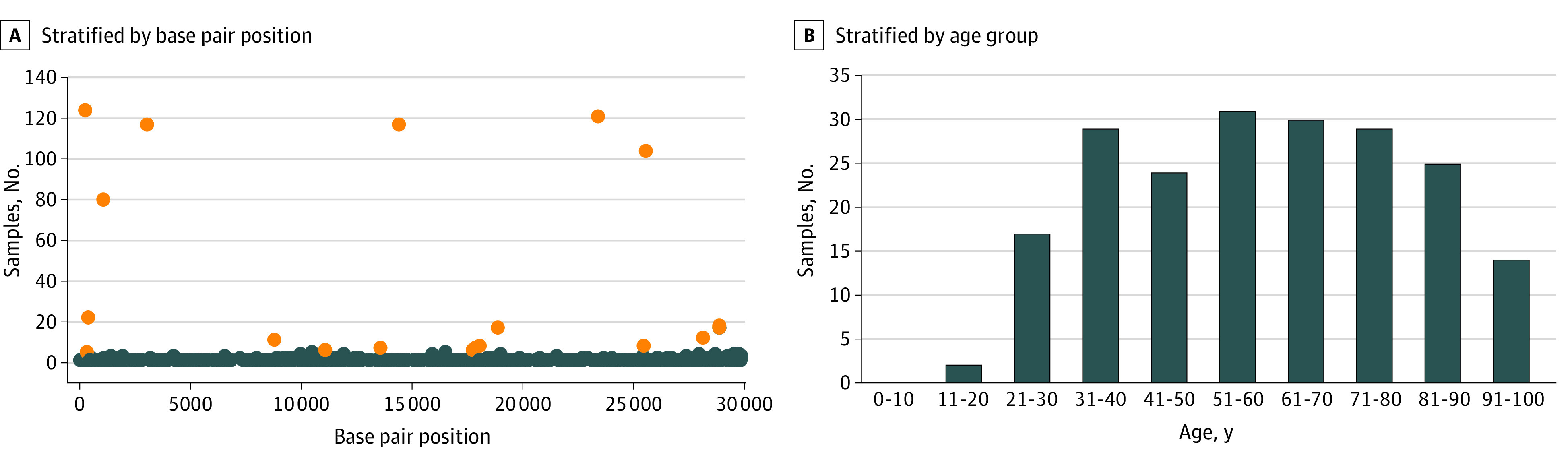
Description of Patient Samples and Severe Acute Respiratory Syndrome Coronavirus 2 Genomic Variations Orange dots indicate the top 20 altered sites; blue dots, the rest of the variations detected.

We sequenced 192 specimens with RT-PCR results positive for SARS-CoV-2 using the Illumina targeted respiratory virus panel. These specimens were collected among 192 patients (median [interquartile range] age, 59.5 [43-75] years; 110 [57.3%] men) (Figure 1). As of May 15, 2020, 21 patients (10.9%) were deceased, 122 patients (63.5%) were admitted and subsequently discharged from the hospital, 11 patients (5.7%) had been admitted and were still hospitalized receiving treatment, and 38 patients (19.8%) were outpatients who had not been hospitalized for COVID-19. The pool of 192 SARS-CoV-2–positive samples obtained 2 222 425 974 reads in raw data (median [interquartile range] mapped reads, 489 759 [152 982-3 172 609]; total reads mapped, 1 737 684 077 reads [78% of total SARS-COV-2 reference genome]). The mapping ratio varied between 0.3% to 99.0%, which negatively correlated with the Ct values obtained from RT-PCR (*R*^2^ = −0.73; *P* < .001). Overall, low mapping ratios with less than 50% genome coverage correlated with samples with increased Ct value (>30 cycles) in the RT-PCR diagnostic test.

### Analyses of Coinfection of Other Respiratory Pathogens and SARS-CoV-2

Sequencing reads from across the sample cohort were mapped to all 41 respiratory viral pathogens (eTable 1 in the Supplement). Despite finding fragmental reads from other viruses, no samples had non–SARS-CoV-2 viral genomes with mapped ratios greater than 5% of total mapped reads in samples with total mapping. Accordingly, there was no evidence of coinfection of other respiratory viral pathogens with SARS-CoV-2 in our sample population.

### Variant Landscape 

Whole-genome comparison of the CSMC samples revealed more than 99.8% identity with the SARS-CoV-2 reference genome. Variation analyses of these isolates revealed a total of 518 variation sites detected across the length of the SARS-CoV-2 genome (Figure 1). A total of 436 variants (84.3%) were private variations and 5 variants (0.1%) were found in more than 50% of all samples (Table). In total, 82 sites had variant in more than 2 isolates containing a mean (SD) of 5.1 (5.0) variants per sample. The top 20 sites with variation and their estimated alterations and frequencies are summarized in the Table and eFigure 1 in the Supplement.

**Table.** Top 20 Alterations of the SARS-CoV-2 Genome Discovered in the Samples Collected From Cedars-Sinai Medical Center^a^

Position	Reference	Alteration	Gene/region	Protein	Amino acid substitution
241	C	T	*5′UTR*	NA	NA
313	C	T	*ORF1ab*	nsp1	Synonymous
379	C	A	*ORF1ab*	nsp1	Synonymous
1059	C	T	*ORF1ab*	nsp2	T>I
3037	C	T	*ORF1ab*	nsp3	Synonymous
8782	C	T	*ORF1ab*	nsp4	Synonymous
11083	G	T	*ORF1ab*	nsp6	L>F
13575	T	C	*ORF1ab*	RdRp	Synonymous
14408	C	T	*ORF1ab*	nsp12	Synonymous
17747	C	T	*ORF1ab*	nsp13	P>L
17858	A	G	*ORF1ab*	Helicase	Y>C
18060	C	T	*ORF1ab*	3′-to-5′exonuclease	Synonymous
18877	C	T	*ORF1ab*	nsp14	Synonymous
23403	A	G	*S*	Spike glycoprotein	D>G
25466	C	T	*ORF3a*	ORF3a protein	P>L
25563	G	T	*ORF3a*	ORF3a protein	Q>H
28144	T	C	*ORF8*	ORF8 protein	L>S
28881	G	A	*ORF9/N*	Nucleocapsid phosphoprotein	R>K
28882	G	A	*ORF9/N*	Nucleocapsid phosphoprotein	R>K^b^
28883	G	C	*ORF9/N*	Nucleocapsid phosphoprotein	G>R

Abbreviation: NA, not available; SARS-CoV-2, severe acute respiratory syndrome coronavirus 2.

^a^ Variation site is depicted in base pairs along the genome sequence of SARS-CoV-2 and estimated the amino acid alteration of the corresponding protein. Main monophyletic clades were labeled based on nucleotide substitutions.

^b^ Amino acid annotation (R>K) is based on the co-occurrence of G28881A and G28882A.

From our most-observed variation sites, 4 variants have been previously reported, including in the 5′-UTR(C241T), along with C3037T, C14408T, and A23403G.^37^ We found 125 samples (65.1%) with all 4 variants present in the genome. While C3037T causes a synonymous variation in nsp3(F105F), C14408T and A23403G resulted in amino acid changes in RNA primase (ie, nsp12, P323L). The China and Northern California variation^10,13^ in the S protein (D614G) was observed in this Los Angeles cohort. Variations at G25563T(ORF3a) and C1059T(nsp2) have been reported to be coexpressed.^37^ The Washington state and China variants,^38^ C8782T(nsp4) and T28144C(ORF8), were also frequently altered in the Los Angeles isolates.

### Phylogenetic Analysis

**Figure 2.**
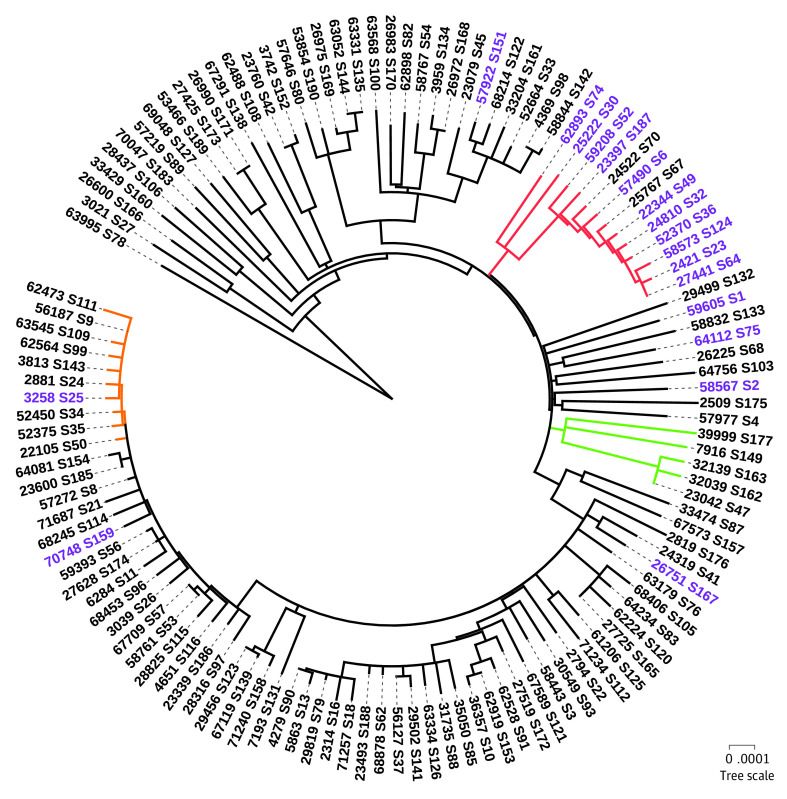
Phylogenetic Tree of Severe Acute Respiratory Syndrome Coronavirus 2 (SARS-CoV-2) Genomes Sampled From Cedars-Sinai Medical Center Patients in Los Angeles, California, Collected From March 22 to April 15, 2020 Red indicates cluster of patients within the same or adjacent postal codes and the same religious denomination; green, cluster of patients with known close contact transmission event; orange, cluster of residents of a skilled nursing facility, health care workers at the facility, a resident of a nearby facility, and a family member of the facility.

**Figure 3.**
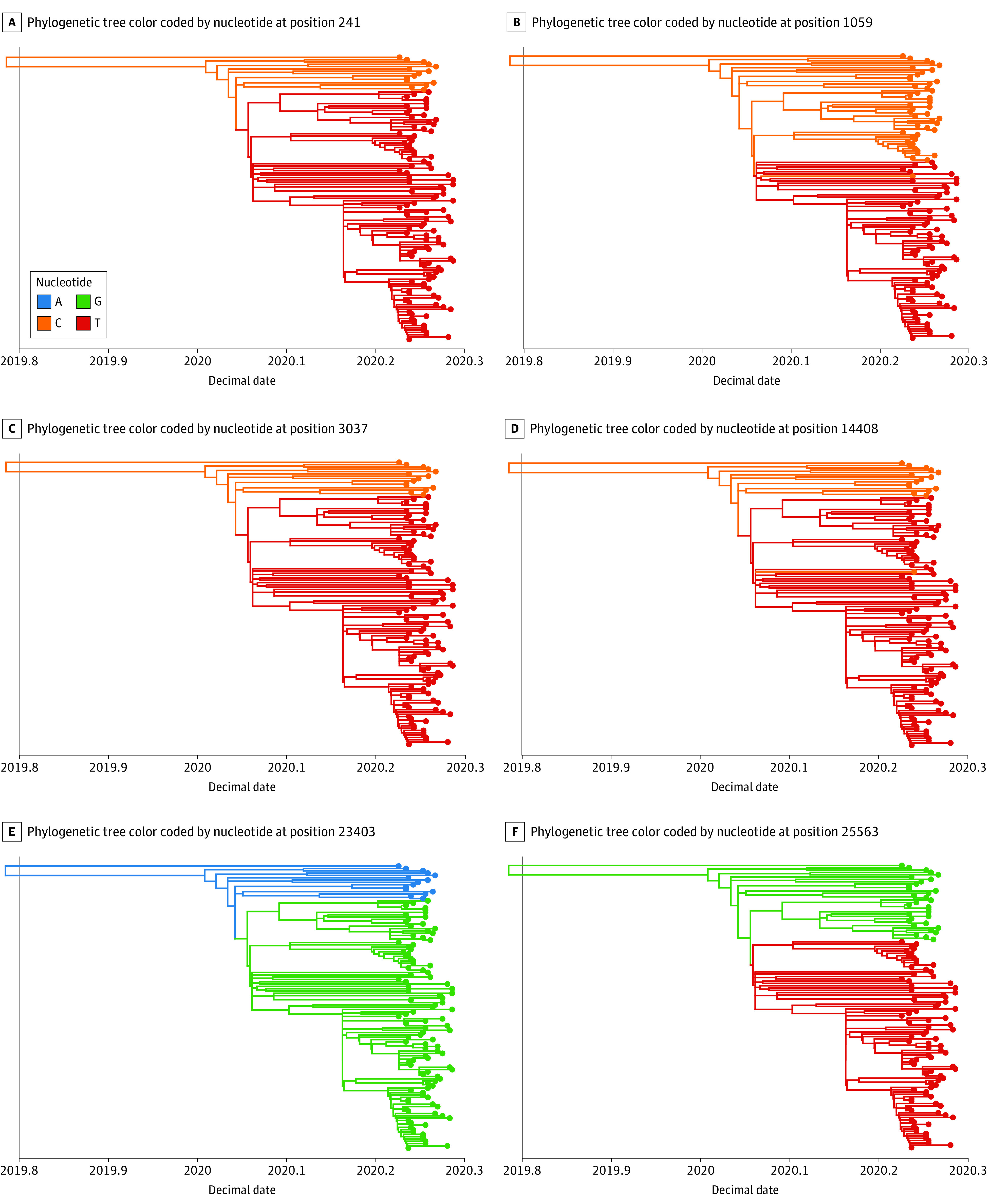
Phylogenetic Tree With the 6 Most Frequently Altered Sites Observed in the Severe Acute Respiratory Syndrome Coronavirus 2 (SARS-CoV-2) Genomes of Cedar-Sinai Medical Center Samples

We performed phylogenetic analysis of 133 samples with more than 50% of the genome covered and more than 10× genome depth to identify which SARS-CoV-2 isolates were most similar (Figure 2). From the top 6 variation sites along the phylogenetic tree (Figure 3), we observed a minimum of 2 groups containing distinct variant signatures. Within these groups, the bottom subclade of the tree contained all 6 variants. A subset of 4 variants that tracked together, as previously described,^37^ were in 2 main clusters (Figure 3A, C, D, and E). While these variants tightly segregated into 2 main clusters of the tree, they did not track with sample collection date (eFigure 2 in the Supplement). The genomic diversity in our population was present from the earliest samples collected and remained throughout the study time frame.

### Phylogenetic Tree Traces of Community Transmission in the Early Stage of the COVID-19 Pandemic

A phylogenetic tree of all Los Angeles isolates was constructed to track SARS-CoV-2 genome differences. A cluster was defined as a group of patients with SARS-CoV-2 strains that originated from the same branching point in the tree. From our local phylogenetic tree analysis, 13 patients, representing more than 10% of our sample population, were identified in 1 cluster (Figure 2). Analysis of the patients’ demographic data revealed that they all lived in the same or adjacent postal codes, within a 3.81 km^2^ radius of each other, and were all members of the same religious denomination. The viral genome exclusively shared between these patients was variant C18877T within the nonstructural protein, nsp14 (eFigure 3 in the Supplement). A community transmission event with known close contact was observed within a tightly associated cluster containing 5 patients, in which all 5 viral genomes shared 3 variants: T13575C, T16506C, and C25466T. Additionally, we observed a cluster of 10 isolates in which 5 patients were known residents of the same skilled nursing facility (SNF) and another patient was a resident of a nearby (ie, within 1 block) SNF. Three additional isolates from this cluster belonged to health care workers with likely contact with patients from the same SNF. The last patient in this cluster was related to one of the patients in the SNF. We did not observe other clear connections within samples outside of these 3 clusters.

### Joint Phylogenetic Analysis

**Figure 4.**
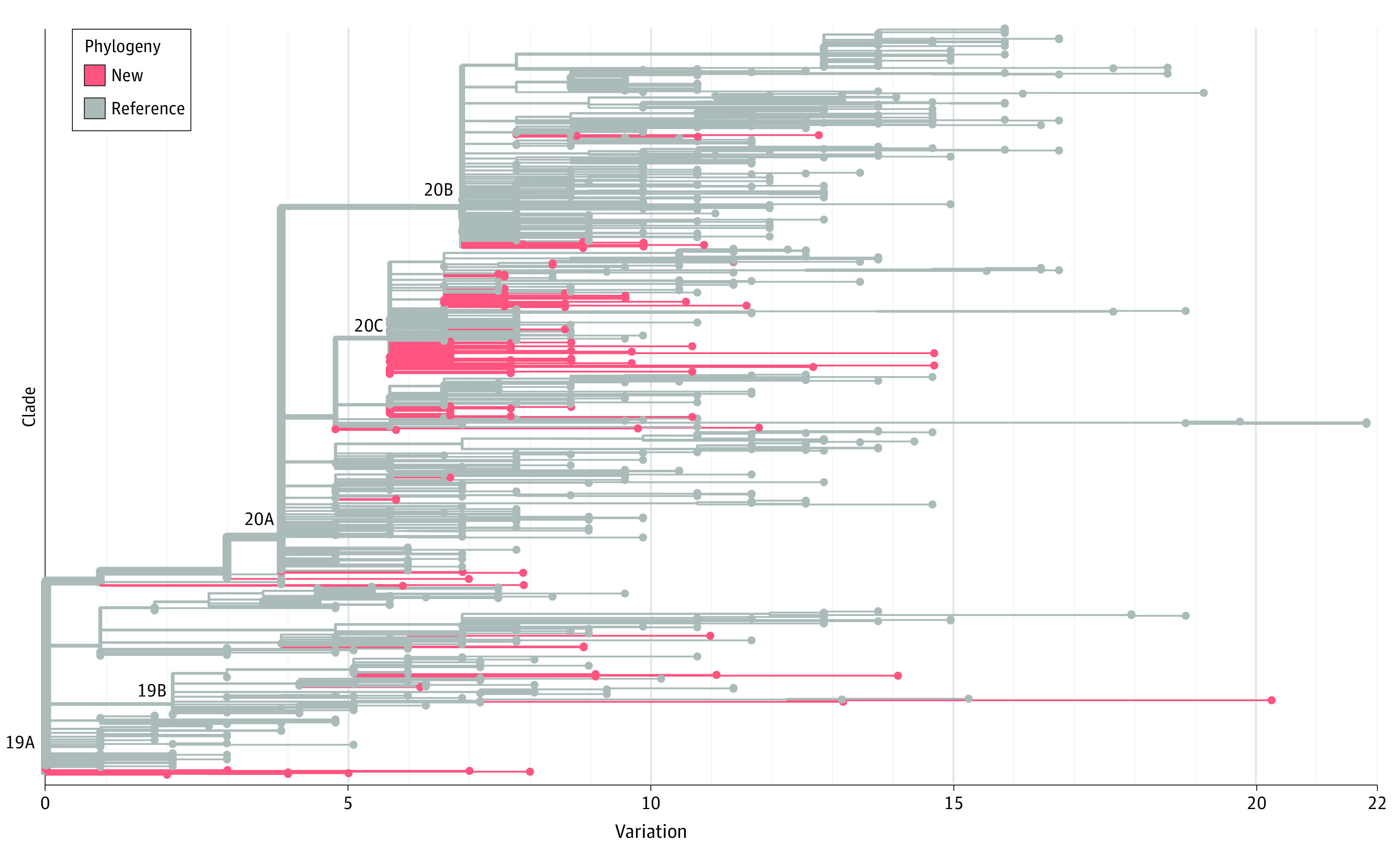
Phylogenetic Tree of Severe Acute Respiratory Syndrome Coronavirus 2 (SARS-CoV-2) Genomes From Los Angeles, California, and a Global Subsampling

To properly address the route of transmission and the distribution of SARS-CoV-2 in the Los Angeles population compared with global distribution of the virus, the CSMC samples were combined with representative genomes subsampled from global data. This phylogenetic tree reveals that the Los Angeles samples were distributed throughout all clades of the SARS-CoV-2 global distribution (Figure 4). The distribution of CSMC samples among these geographically distributed isolates is indicative of multiple independent viral introductions into the Los Angeles community. Among the 2 major clades, 20 (15.0%) were similar to the Asian lineage and 109 (82.0%) were similar to the European lineages. More than half of the CSMC SARS-CoV-2 genomes (72 samples [54.1%]) were within clade 20C, which contains predominantly North America isolates. Additionally, 24 CSMC isolates (18.0%) were in clade 20A, which contains mainly early European isolates. There are 2 main clusters (clade 19A and subclade19B) from Asia (mainly China) in which CSMC samples were found in both groups, with 13 samples (9.7%) in clade 19A and 7 samples (5.3%) in clade 19B. Clade 20B contains 13 isolates (9.7%) that clustered with another Europe-originating clade, distinguished by 3 consecutive variants: G28881A, G28882A, and G28883C. An unknown clade, including 4 isolates (3.0%), is consistent with the emerging global tree. Phylogenetic analyses of the Los Angeles isolates with genomes from New York, Washington state, and China found that they shared similarities to all subclades derived from these regional locations (eFigure 4 in the Supplement).

## Discussion

To our knowledge, this case series is the first comprehensive study of a COVID-19 sample population from Los Angeles, one of the major outbreak centers in the US. A caveat to our sample collection is that emergency departments are less frequented by younger patients and biased to patients 18 years and older. Thus, the mean age of CSMC patients was approximately 60 years, which is consistent with older adults being more susceptible to COVID-19.^5,21,24^ Patients with higher viral loads detected by RT-PCR also correlated with a higher percentage of SARS-CoV-2 genome coverage by sequencing. From a technical perspective, 48 patients with lower sequencing coverage (less than 50% of the total cohort) were diagnostically confirmed to have SARS-CoV-2 infection by RT-PCR testing at more than 30 cycles.^39^ Thus, when using NGS approaches for diagnostic purposes, a potential caveat is that genome sequencing favors patients with higher viral titers and may not capture those who have low viral copy numbers.

Analysis of 40 other respiratory viruses did not reveal coinfection with SARS-CoV-2 in our cohort, which is consistent with other studies, indicating that rates of coinfection are low in patients with SARS-CoV-2 infection.^40^ However, we could not rule out the possibility of coinfection or superinfection for viruses with low copy numbers but the high viral load of SARS-CoV-2 made it preferentially sequenced. As knowledge of this virus is rapidly evolving, these data become important in helping the greater medical community understand the variability of presentation of SARS-CoV-2 with other viral pathogens.

The local phylogenetic tree found 2 large clusters, which were mainly defined by 6 high-frequency variations. Phylogenetic analysis of these samples by collection date reveals that the main variants that defined these 2 large clusters were observed throughout March and April; therefore, they were present in the community prior to our collection date,

This case series presents a snapshot of the molecular characteristics of SARS-CoV-2 early transmission into the Los Angeles area. The window of our collection dates was not long enough to observe new viral dissemination data into the Los Angeles population. Despite our local phylogenetic tree showing high genomic diversity, tight clustering patterns within a group of 5 patients was detected from their genomes sharing 1 variant in common. This finding highlights the precision of contact tracing directly through SARS-CoV-2 genome isolation and sequencing, by which genomic analysis of this variant can precisely track person-to-person transmission within a larger urban area.

Another unique cluster in the local phylogenetic tree found a cluster of patients who were identified within the same or adjacent postal code. This postal code is only 3.81 km^2^ and densely populated (36 885 people). This cluster represents spread within a constrained geographic area all within members of the same religious community. Further validating this representative spread within a distinct community was the fact that the SARS-CoV-2 isolates of 7 patients from the same postal code who were not from the same religious denomination were not found in this cluster. Previous studies highlight religious communities being at particularly acute risk in a pandemic owing to large communal events, such as services, weddings, and funerals.^19,41^ Moving forward, community leaders should be aware of the unique risks posed to their congregations and plan accordingly. The remaining patients lived across many postal codes, providing further evidence of community transmission across the larger metropolitan area.

A third cluster showed widespread transmission within a single SNF. Such facilities have been a hotbed for viral spread worldwide, and it is not surprising to observe this type of clustering.

Global initiatives to track SARS-CoV-2 have proven fruitful in monitoring disease incidence, severity, and worldwide spread.^6,9,11,12,13,14,18,42,43,44,45,46,47,48,49^ In this study, by examining a cohort within a SARS-CoV-2 US epicenter, Los Angeles, we lay the foundation for further studies into the use of SARS-CoV-2 sequencing to monitor local community spread.

### Limitations

This study has some limitations, including that SARS-CoV-2 genomes were all from patients who were hospitalized for COVID-19 and may be a biased representation of more severe cases. These samples were obtained early during the US pandemic, when testing was limited, and a high proportion of individuals with asymptomatic infection or mild symptoms are absent in this and similar studies.^46,50^ These missing SARS-CoV-2 infections will affect the collective assessment of transmission both in the US and globally. When attempting to infer causality, Villabona-Arenas et al^51^ provided examples of pitfalls that can occur by performing epidemiological analysis on viral genomes alone, especially when the virus is novel. The possibility remains that multiple seed events in Los Angeles, Europe, and New York occurred simultaneously, thus confounding the ability to draw directionality from the data. Considering the timing of the COVID-19 spread and the known transmission patterns from Europe to New York, we consider this unlikely. What may be more plausible, and should be considered, is that travelers from Europe seeded New York and Los Angeles simultaneously. Lu et al^18^ also highlight how phylogenetic analysis can be misleading, as clusters thought to represent community spread can include multiple introductions from genomically undersampled locations. Their study was biased by the fact that data were collected primarily during the spring festival period surrounding the Chinese New Year, the period of largest annual human migration event in the world.^52^ Expectedly, a significantly larger portion of cases than normal were imported from outside regions. There was no such event in Los Angeles at the time of the early outbreak, and the data in this study were generated several weeks after state-ordered limitations on travel and gatherings had been enacted. Although we have a limited sample number (133 patients), the integration of CSMC SARS-CoV-2 genomes into Washington state, New York City and China (eFigure 4 in the Supplement) data sets, provided helpful insight into determining the introduction of SARS-CoV-2 into the Los Angeles community.

## Conclusions

In this case series, consistent with other studies, the combination of the 4 variants (ie, C241T, C3037T, C14408T, and A23403G) coevolving together has been seen in other tracked populations in European isolates.^9,37^ From our variant analysis, 2 of our highly altered sites, G25563T(ORF3a) and C1059T(nsp2), have been reported exclusively in US isolated sequences collected since March 2020,^7^ a timeline that corresponds to this study’s sample collection date. These variants were found to be closely associated within a cluster containing mainly SARS-CoV-2 genomes from New York, suggesting that these genomes were introduced from a strain that emerged from the US East Coast population. From the variants found in our samples, 4 variants, 5′-UTR (241C>T), 3037C>T, 14408C>T, and 23403A>G, agree with other studies that found that these variations coevolved.^37^ Such a high proportion of our patients having all 4 variation indicates the seeding of our population by a strain originating in Europe. This finding is further validated in our local phylogenetic tree, which separates into 2 main clusters, our global tree in which our population closely resembles SARS-CoV-2 genomes from New York,^9^ followed by a smaller percentage from Washington state, together identifying possible routes for the dissemination of SARS-CoV-2 into the Southern California populace. Given that Seattle, Washington, was the first documented US appearance of SARS-CoV-2, the introduction of the virus from Washington state^13,20^ is consistent with our phylogenetic tree and the time frame of our data sampling, concordant with our hypothesis. However, despite our earlier estimates, an even larger portion of our sample population had a significant resemblance to genomes from New York, the epicenter of the SARS-CoV-2 outbreak in the US.^9,12,44^ The appearance of the majority of our samples within different subclades of New York isolates suggests that SARS-CoV-2 likely spread from multiple introductions from New York. Furthermore, the CSMC population interspersed with Washington state and China isolates suggests multiple dissemination routes from Asia and the US Northern West Coast to Southern California, appearing as a major cluster in our local population. Although we restricted our analyses to these 3 geographical origins, we found high genomic diversity among the CSMC SARS-CoV-2 isolates. The large impact of COVID-19 on the Los Angeles community likely originated from independent disseminations of the virus from multiple routes, with some geographical strains having greater prevalence than others.

## Notes

Supplement. **eFigure 1.** Frequency of the Top 20 Altered Sites Found in SARS-CoV-2 Genomes Sampled From Cedars-Sinai Medical Center Patients in Los Angeles, California **eFigure 2.** Phylogenetic Tree Scaled 133 Isolates Collected From Cedars-Sinai Medical Center Based on the Date of Collection **eFigure 3.** Phylogenetic Tree Clade Labelling the Variant C18877T in the SARS-CoV-2 Genome of 133 Isolates Collected From Cedars Sinai Medical Center **eFigure 4.** Phylogenetic Tree of 3343 SARS-CoV-2 Genomes **eTable 1.** Respiratory Viruses Included in the Sequencing Panel **eTable 2.** GISAID Deposited Genomes and Their Accession Numbers for the Isolates Used in this Study **eTable 3.** Accession Number for GISAID SARS-CoV-2 Genomes Collected From Washington State; New York, New York; and China Analyzed With Integration of CSMC Samples
