# Supplementary material for: Analysis of Genomic Characteristics and Transmission Routes of Patients With Confirmed SARS-CoV-2 in Southern California During the Early Stage of the US COVID-19 Pandemic
Source: JAMA Netw Open. 2020 Oct 7;3(10):e2024191. doi: 10.1001/jamanetworkopen.2020.24191 (PMC7542329; doi:10.1001/jamanetworkopen.2020.24191)
Supplement: Supplement. — eFigure 1. Frequency of the Top 20 Altered Sites Found in SARS-CoV-2 Genomes Sampled From Cedars-Sinai Medical Center Patients in Los Angeles, California eFigure 2. Phylogenetic Tree Scaled 133 Isolates Collected From Cedars-Sinai Medical Center Based on the Date of Collection eFigure 3. Phylogenetic Tree Clade Labelling the Variant C18877T in the SARS-CoV-2 Genome of 133 Isolates Collected From Cedars Sinai Medical Center eFigure 4. Phylogenetic Tree of 3343 SARS-CoV-2 Genomes eTable 1. Respiratory Viruses Included in the Sequencing Panel eTable 2. GISAID Deposited Genomes and Their Accession Numbers for the Isolates Used in this Study eTable 3. Accession Number for GISAID SARS-CoV-2 Genomes Collected From Washington State; New York, New York; and China Analyzed With Integration of CSMC Samples [file jamanetwopen-e2024191-s001.pdf]

Supplemental Online Content

Zhang W, Govindavari JP, Davis BD, et al. Analysis of genomic characteristics and transmission routes of patients with confirmed SARS-CoV-2 in Southern California during the early stage of the US COVID-19 pandemic. *JAMA Netw Open*. 2020;3(10):e2024191. doi:10.1001/jamanetworkopen.2020.24191

eFigure 1. Frequency of the Top 20 Altered Sites Found in SARS-CoV-2 Genomes Sampled From Cedars-Sinai Medical Center Patients in Los Angeles, California

eFigure 2. Phylogenetic Tree Scaled 133 Isolates Collected From Cedars-Sinai Medical Center Based on the Date of Collection

eFigure 3. Phylogenetic Tree Clade Labelling the Variant C18877T in the SARS-CoV-2 Genome of 133 Isolates Collected From Cedars Sinai Medical Center

eFigure 4. Phylogenetic Tree of 3343 SARS-CoV-2 Genomes

eTable 1. Respiratory Viruses Included in the Sequencing Panel

eTable 2. GISAID Deposited Genomes and Their Accession Numbers for the Isolates Used in this Study

eTable 3. Accession Number for GISAID SARS-CoV-2 Genomes Collected From Washington State; New York, New York; and China Analyzed With Integration of CSMC Samples

This supplemental material has been provided by the authors to give readers additional information about their work.

© 2020 Zhang W et al. *JAMA Network Open.*

eFigure 1. Frequency of the Top 20 Altered Sites Found in SARS-CoV-2 Genomes Sampled From Cedars-Sinai Medical Center Patients in Los Angeles, California

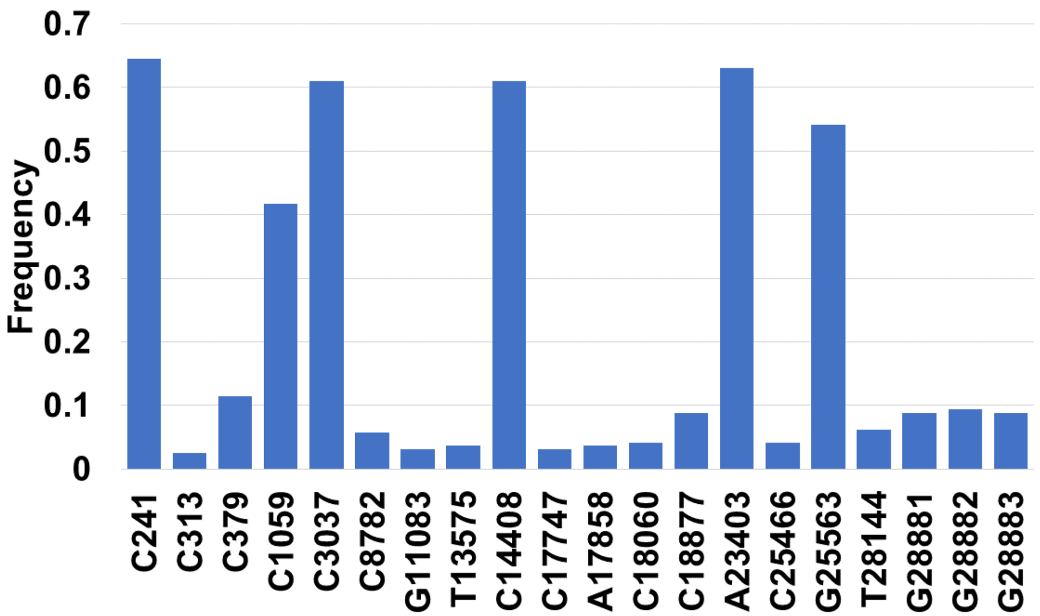

Histogram displays the nucleotide changes observed and the occurrence within all samples.

© 2020 Zhang W et al. *JAMA Network Open.*

eFigure 2. Phylogenetic Tree Scaled 133 Isolates Collected From Cedars-Sinai Medical Center Based on the Date of Collection

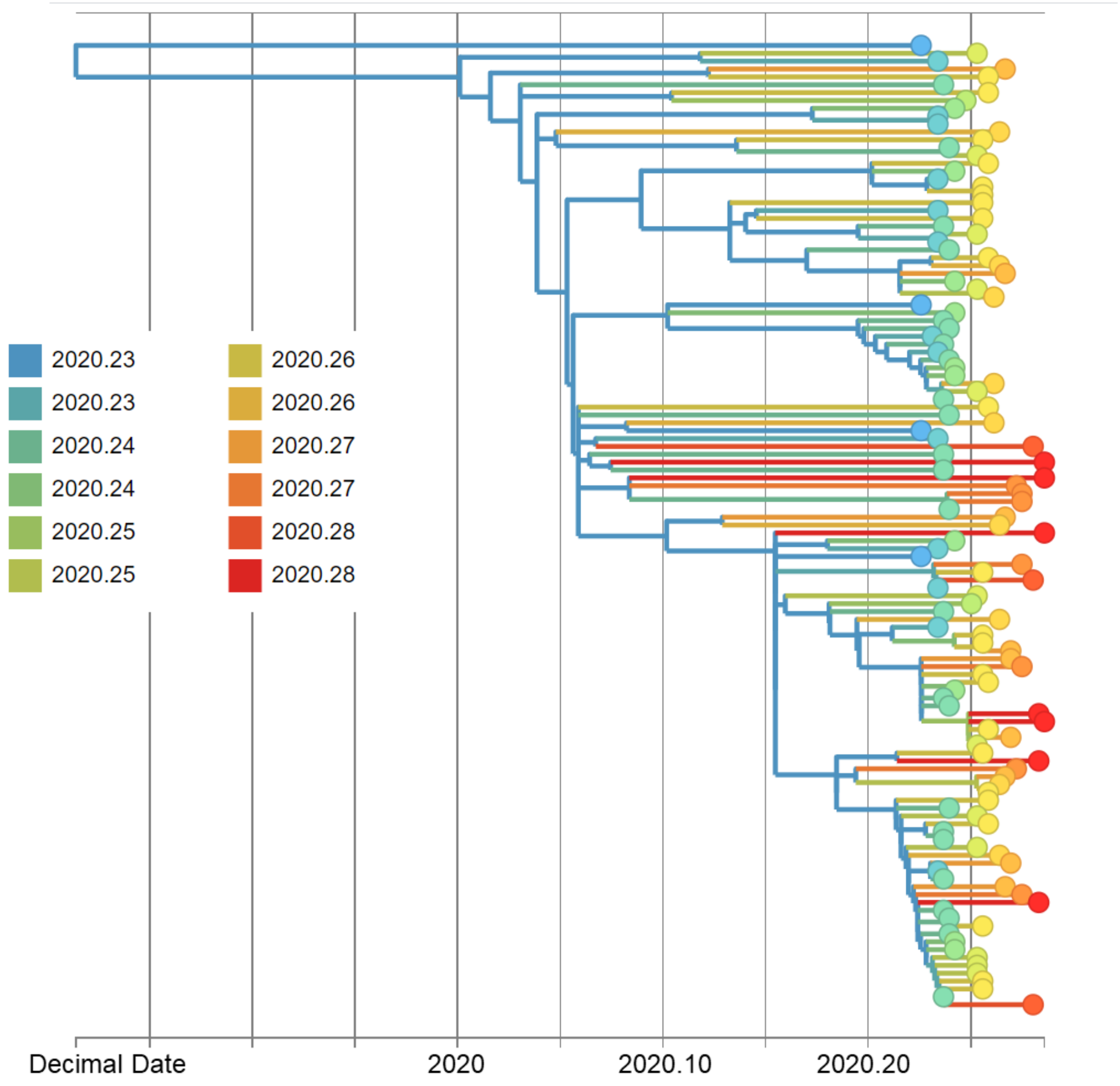

© 2020 Zhang W et al. *JAMA Network Open.*

eFigure 3. Phylogenetic Tree Clade Labelling the Variant C18877T in the SARS-CoV-2 Genome of 133 Isolates Collected From Cedars Sinai Medical Center

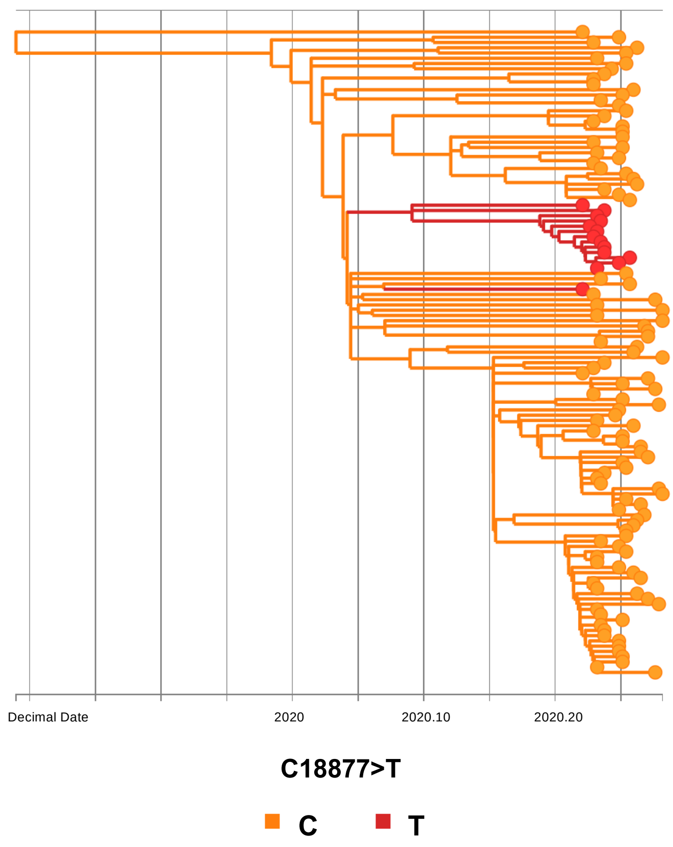

Histogram depicts one isolated cluster in which only those isolates contain this variant change.

© 2020 Zhang W et al. *JAMA Network Open.*

eFigure 4. Phylogenetic Tree of 3343 SARS-CoV-2 Genomes

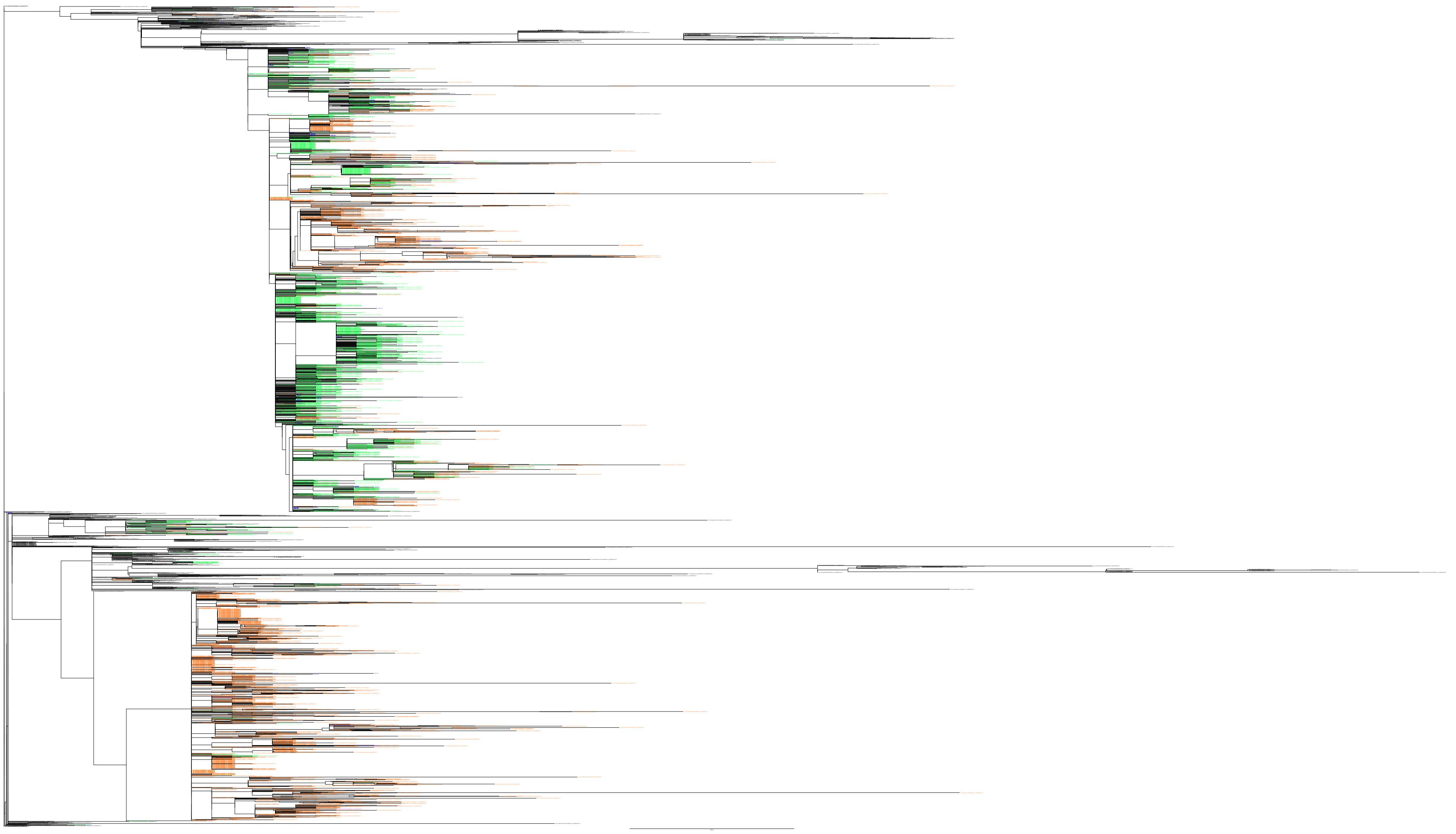

This tree combines SARS-CoV-2 genomes deposited to GISAID from Washington state(orange), New York City (green) and China (black) to those sequenced from Cedars-Sinai Medical Center patients in Los Angeles, USA (blue). Clusters (A-G) are labelled by their similarities within geographic isolates.

© 2020 Zhang W et al. *JAMA Network Open.*

eTable 1. Respiratory Viruses Included in the Sequencing Panel

Name	Accession	Length(bp)
Human parechovirus 6	AB252582	7347
Human enterovirus C104 strain: AK11	AB686524	7408
Human Respiratory syncytial virus 9320 (type B)	AY353550	15225
Human coronavirus OC43	AY391777	30738
Influenza B virus (B/Wisconsin/01/2010)	CY115156.1	2245
Influenza B virus (B/Wisconsin/01/2010)	CY115183.1	1789
Influenza B virus (B/Wisconsin/01/2010)	CY115184.1	1151
Influenza B virus (B/Wisconsin/01/2010)	CY115185.1	1512
Influenza B virus (B/Wisconsin/01/2010)	CY115186.1	1762
Influenza B virus (B/Wisconsin/01/2010)	CY115187.1	1063
Influenza B virus (B/Wisconsin/01/2010)	CY115188.1	2255
Influenza B virus (B/Wisconsin/01/2010)	CY115189.1	2330
Influenza B virus (B/Wisconsin/01/2010)	CY115190.1	2358
Influenza B virus (B/Colorado/06/2017)	CY236610.1	1154
Influenza B virus (B/Brisbane/60/2008)	FJ766839.1	1557
Human parechovirus type 1 PicoBank/HPeV1/a	FM242866	7296
Human enterovirus C109 isolate NICA08-4327	GQ865517	7354
Influenza B virus (B/Brisbane/60/2008)	KC866603.1	2369
Influenza B virus (B/Brisbane/60/2008)	KC866604.1	2396
Influenza B virus (B/Brisbane/60/2008)	KC866605.1	1844
Influenza B virus (B/Brisbane/60/2008)	KC866606.1	1101
Influenza B virus (B/Brisbane/60/2008)	KC866607.1	1189
Influenza A virus (A/Zhejiang/DTID-ZJU01/2013(H7N9))	KC885955	2209
Influenza A virus (A/Zhejiang/DTID-ZJU01/2013(H7N9))	KC885956	1710
Influenza A virus (A/Zhejiang/DTID-ZJU01/2013(H7N9))	KC885957	1520
Influenza A virus (A/Zhejiang/DTID-ZJU01/2013(H7N9))	KC885958	1426
Influenza A virus (A/Zhejiang/DTID-ZJU01/2013(H7N9))	KC885959	1002
Influenza A virus (A/Zhejiang/DTID-ZJU01/2013(H7N9))	KC885960	864
Influenza A virus (A/Zhejiang/DTID-ZJU01/2013(H7N9))	KC885961	2317
Influenza A virus (A/Zhejiang/DTID-ZJU01/2013(H7N9))	KC885962	2314
Influenza A virus (A/Texas/50/2012(H3N2))	KJ942616.1	1749
Influenza A virus (A/Texas/50/2012(H3N2))	KJ942617.1	1016
Influenza A virus (A/Texas/50/2012(H3N2))	KJ942618.1	1462
Influenza A virus (A/Texas/50/2012(H3N2))	KJ942619.1	1555
Influenza A virus (A/Texas/50/2012(H3N2))	KJ942620.1	875
Influenza A virus (A/Texas/50/2012(H3N2))	KJ942621.1	2222
Influenza A virus (A/Texas/50/2012(H3N2))	KJ942622.1	2330
Influenza A virus (A/Texas/50/2012(H3N2))	KJ942623.1	2333

© 2020 Zhang W et al. *JAMA Network Open.*

Influenza A virus (A/Michigan/45/2015(H1N1))	KU509700.1	2280
Influenza A virus (A/Michigan/45/2015(H1N1))	KU509701.1	2274
Influenza A virus (A/Michigan/45/2015(H1N1))	KU509702.1	2151
Influenza A virus (A/Michigan/45/2015(H1N1))	KU509703.1	1701
Influenza A virus (A/Michigan/45/2015(H1N1))	KU509704.1	1497
Influenza A virus (A/Michigan/45/2015(H1N1))	KU509705.1	1410
Influenza A virus (A/Michigan/45/2015(H1N1))	KU509706.1	982
Influenza A virus (A/Michigan/45/2015(H1N1))	KU509707.1	863
Influenza B virus (B/Brisbane/60/2008)	KX058884.1	1885
Influenza B virus (B/Washington/02/2019)	MN325123.1	2276
SARS-CoV-2	MN908947.3	29903
Human adenovirus C2	NC_001405	35937
Human rhinovirus B14	NC_001490	7212
Human rhinovirus A89	NC_001617	7152
Human parainfluenza virus 3	NC_001796	15462
Respiratory syncytial virus (type A)	NC_001803	15191
Influenza A virus (A/Puerto Rico/8/1934(H1N1))	NC_002016	1027
Influenza A virus (A/Puerto Rico/8/1934(H1N1))	NC_002017	1778
Influenza A virus (A/Puerto Rico/8/1934(H1N1))	NC_002018	1413
Influenza A virus (A/Puerto Rico/8/1934(H1N1))	NC_002019	1565
Influenza A virus (A/Puerto Rico/8/1934(H1N1))	NC_002020	890
Influenza A virus (A/Puerto Rico/8/1934(H1N1))	NC_002021	2341
Influenza A virus (A/Puerto Rico/8/1934(H1N1))	NC_002022	2233
Influenza A virus (A/Puerto Rico/8/1934(H1N1))	NC_002023	2341
Influenza B virus (B/Lee/1940)	NC_002204	2368
Influenza B virus (B/Lee/1940)	NC_002205	2313
Influenza B virus (B/Lee/1940)	NC_002206	2204
Influenza B virus (B/Lee/1940)	NC_002207	1882
Influenza B virus (B/Lee/1940)	NC_002208	1841
Influenza B virus (B/Lee/1940)	NC_002209	1557
Influenza B virus (B/Lee/1940)	NC_002210	1191
Influenza B virus (B/Lee/1940)	NC_002211	1096
Human coronavirus 229E	NC_002645	27317
Human adenovirus E4	NC_003266	35994
Human parainfluenza virus 2	NC_003443	15646
Human parainfluenza virus 1	NC_003461	15600
Human metapneumovirus (CAN97-83)	NC_004148	13335
Influenza A virus (A/Hong Kong/1073/99(H9N2))	NC_004905	1557
Influenza A virus (A/Hong Kong/1073/99(H9N2))	NC_004906	890
Influenza A virus (A/Hong Kong/1073/99(H9N2))	NC_004907	1025
Influenza A virus (A/Hong Kong/1073/99(H9N2))	NC_004908	1714

© 2020 Zhang W et al. *JAMA Network Open.*

Influenza A virus (A/Hong Kong/1073/99(H9N2))	NC_004909	1418
Influenza A virus (A/Hong Kong/1073/99(H9N2))	NC_004910	2341
Influenza A virus (A/Hong Kong/1073/99(H9N2))	NC_004911	2328
Influenza A virus (A/Hong Kong/1073/99(H9N2))	NC_004912	2225
Human coronavirus NL63	NC_005831	27553
Human coronavirus HKU1	NC_006577	29926
Influenza A virus (A/goose/Guangdong/1/1996(H5N1))	NC_007357	2341
Influenza A virus (A/goose/Guangdong/1/1996(H5N1))	NC_007358	2341
Influenza A virus (A/goose/Guangdong/1/1996(H5N1))	NC_007359	2233
Influenza A virus (A/goose/Guangdong/1/1996(H5N1))	NC_007360	1565
Influenza A virus (A/goose/Guangdong/1/1996(H5N1))	NC_007361	1458
Influenza A virus (A/goose/Guangdong/1/1996(H5N1))	NC_007362	1760
Influenza A virus (A/goose/Guangdong/1/1996(H5N1))	NC_007363	1027
Influenza A virus (A/goose/Guangdong/1/1996(H5N1))	NC_007364	865
Influenza A virus (A/New York/392/2004(H3N2))	NC_007366	1762
Influenza A virus (A/New York/392/2004(H3N2))	NC_007367	1027
Influenza A virus (A/New York/392/2004(H3N2))	NC_007368	1467
Influenza A virus (A/New York/392/2004(H3N2))	NC_007369	1566
Influenza A virus (A/New York/392/2004(H3N2))	NC_007370	890
Influenza A virus (A/New York/392/2004(H3N2))	NC_007371	2233
Influenza A virus (A/New York/392/2004(H3N2))	NC_007372	2341
Influenza A virus (A/New York/392/2004(H3N2))	NC_007373	2341
Influenza A virus (A/Korea/426/1968(H2N2))	NC_007374	1773
Influenza A virus (A/Korea/426/1968(H2N2))	NC_007375	2341
Influenza A virus (A/Korea/426/1968(H2N2))	NC_007376	2233
Influenza A virus (A/Korea/426/1968(H2N2))	NC_007377	1027
Influenza A virus (A/Korea/426/1968(H2N2))	NC_007378	2341
Influenza A virus (A/Korea/426/1968(H2N2))	NC_007380	838
Influenza A virus (A/Korea/426/1968(H2N2))	NC_007381	1497
Influenza A virus (A/Korea/426/1968(H2N2))	NC_007382	1410
Human bocavirus 1 (Primate bocaparvovirus 1 isolate st2)	NC_007455	5299
KI polyomavirus Stockholm 60	NC_009238	5040
WU Polyomavirus	NC_009539	5229
Human rhinovirus C (strain 024)	NC_009996	7099
Human adenovirus B1	NC_011203	35343
Human bocavirus 2c PK isolate PK-5510	NC_012042	5196
Human bocavirus 3	NC_012564	5242
Human bocavirus 4 NI strain HBoV4-NI-385	NC_012729	5104
Human parainfluenza virus 4a	NC_021928	17052

© 2020 Zhang W et al. *JAMA Network Open.*

eTable 2. GISAID Deposited Genomes and Their Accession Numbers for the Isolates Used in this Study

Sample ID	GISAID Accession	Pangolin lineage	NextStrain
22105_S50	EPI_ISL_467809	B.1	20C
22344_S49	EPI_ISL_475629	B.1	20A
23042_S47	EPI_ISL_475640	B.1	20C
23079_S45	EPI_ISL_475651	B.1.1	20B
2314_S16	EPI_ISL_475662	B.1.43	20C
23339_S186	EPI_ISL_475673	B.1	20C
23397_S187	EPI_ISL_475684	B.1	20A
23493_S188	EPI_ISL_475695	B.1.43	20C
23600_S185	EPI_ISL_475706	B.1	20C
23760_S42	EPI_ISL_475574	B.4	19A
2421_S23	EPI_ISL_475585	B.1	20A
24319_S41	EPI_ISL_475596	B.1	20C
24522_S70	EPI_ISL_475618	B.1	20A
24810_S32	EPI_ISL_475624	B.1	20A
2509_S175	EPI_ISL_475625	B.1	-
25222_S30	EPI_ISL_475626	B.1	20A
25767_S67	EPI_ISL_475627	B.1	20A
26225_S68	EPI_ISL_475628	B.1	20C
26600_S166	EPI_ISL_475630	A.1	19B
26751_S167	EPI_ISL_475631	B.1	20C
26972_S168	EPI_ISL_475632	B.1.1	20B
26975_S169	EPI_ISL_475633	B.1	20A
26983_S170	EPI_ISL_475634	B.1.1	20B
26990_S171	EPI_ISL_475635	B	19A
27425_S173	EPI_ISL_475636	B	19A
27441_S64	EPI_ISL_475637	B.1	20A
27519_S172	EPI_ISL_475638	B.1.43	20C
27628_S174	EPI_ISL_475639	B.1	20C
27725_S165	EPI_ISL_475641	B.1.3	20C
2794_S22	EPI_ISL_475642	B.1.43	20A
2819_S176	EPI_ISL_475643	B.1	20C
28316_S97	EPI_ISL_475644	B.1	20C
28437_S106	EPI_ISL_475645	A.1	19B
2881_S24	EPI_ISL_475646	B.1	20C
28825_S115	EPI_ISL_475647	B.1.2	20C
29456_S123	EPI_ISL_475648	B.1	20C
29499_S132	EPI_ISL_475649	B.1	20A
29502_S141	EPI_ISL_475650	B.1.43	20C
29819_S79	EPI_ISL_475652	B.1.43	20C
29956_S84	EPI_ISL_475653	B.1	20A
3021_S27	EPI_ISL_475654	A.1	19B
3039_S26	EPI_ISL_475655	B.1	20A
30549_S93	EPI_ISL_475656	B.1.43	20C
31138_S110	EPI_ISL_475657	B.1	20A

© 2020 Zhang W et al. *JAMA Network Open.*

31735_S88	EPI_ISL_475658	B.1.43	20C
32039_S162	EPI_ISL_475659	B.1	20C
32139_S163	EPI_ISL_475660	B.1	20C
3258_S25	EPI_ISL_475661	B.1	20C
33204_S161	EPI_ISL_475663	B.1.1	20B
33429_S160	EPI_ISL_475664	A.1	19B
33474_S87	EPI_ISL_475665	B.1	20C
35050_S85	EPI_ISL_475667	B.1.43	20C
36357_S10	EPI_ISL_475668	B.1.43	20C
3742_S152	EPI_ISL_475669	B.4	19A
3813_S143	EPI_ISL_475670	B.1	20C
3959_S134	EPI_ISL_475671	B.1.1	20B
39999_S177	EPI_ISL_475672	B.1	19A
4279_S90	EPI_ISL_475674	B.1.43	20C
4369_S98	EPI_ISL_475675	B.1.1	20B
4651_S116	EPI_ISL_475676	B.1	20C
52370_S36	EPI_ISL_475677	B.1	20A
52375_S35	EPI_ISL_475678	B.1	20C
52450_S34	EPI_ISL_475679	B.1	20C
52664_S33	EPI_ISL_475680	B.1.1	20B
53466_S189	EPI_ISL_475681	B	19A
53854_S190	EPI_ISL_475682	B.1	20A
56127_S37	EPI_ISL_475683	B.1.43	20C
56187_S9	EPI_ISL_475685	B.1	20C
57219_S89	EPI_ISL_475686	A.3	19B
57272_S8	EPI_ISL_475687	B.1	20C
57413_S7	EPI_ISL_475688	B.1	20C
57490_S6	EPI_ISL_475689	B.1	20A
57646_S80	EPI_ISL_475690	B.1	20A
57922_S151	EPI_ISL_475691	B.1.1	20B
57933_S5	EPI_ISL_475692	B.1	20C
57977_S4	EPI_ISL_475693	B.1	19A
58443_S3	EPI_ISL_475694	B.1.43	20C
58567_S2	EPI_ISL_475696	B.1	-
58573_S124	EPI_ISL_475697	B.1	20A
5863_S13	EPI_ISL_475698	B.1.43	20C
58761_S53	EPI_ISL_475699	B.1.2	20C
58767_S54	EPI_ISL_475700	B.1.1	20B
58832_S133	EPI_ISL_475701	B.1	-
58844_S142	EPI_ISL_475702	B.1.1	20B
59208_S52	EPI_ISL_475703	B.1	20A
59393_S56	EPI_ISL_475705	B.1	20C
59605_S1	EPI_ISL_475707	B.1	19A
61206_S125	EPI_ISL_475709	B.1	20C
62224_S120	EPI_ISL_475710	B.1.3	20C
62473_S111	EPI_ISL_475711	B.1	20C
62488_S108	EPI_ISL_475712	B.4	19A
62528_S91	EPI_ISL_475713	B.1.43	20C

© 2020 Zhang W et al. *JAMA Network Open.*

62564_S99	EPI_ISL_475714	B.1	20C
6284_S11	EPI_ISL_475715	B.1	20C
62893_S74	EPI_ISL_475716	B.1	20A
62898_S82	EPI_ISL_475575	B.1.1	19A
62919_S153	EPI_ISL_475576	B.1.43	20C
63052_S144	EPI_ISL_475577	B.1	20A
63179_S76	EPI_ISL_475578	B.1	20C
63331_S135	EPI_ISL_475579	B.1	20A
63334_S126	EPI_ISL_475580	B.1.43	20C
63545_S109	EPI_ISL_475582	B.1	20C
63568_S100	EPI_ISL_475583	B.1.1	20B
63995_S78	EPI_ISL_475586	A.1	-
64081_S154	EPI_ISL_475588	B.1	20C
64112_S75	EPI_ISL_475589	B.1	19A
64234_S83	EPI_ISL_475590	B.1.3	20C
64756_S103	EPI_ISL_475591	B.1	20C
67119_S139	EPI_ISL_475594	B.1	20C
67291_S138	EPI_ISL_475597	B	19A
67573_S157	EPI_ISL_475598	B.1	20C
67589_S121	EPI_ISL_475599	B.1.43	20C
67709_S57	EPI_ISL_475600	B.1.2	20C
68214_S122	EPI_ISL_475601	B.1.1	20B
68234_S113	EPI_ISL_475602	B.1.1	20B
68245_S114	EPI_ISL_475603	B.1	20C
68309_S156	EPI_ISL_475604	B.1	20C
68406_S105	EPI_ISL_475605	B.1.3	20C
68453_S96	EPI_ISL_475606	B.1	20C
68511_S63	EPI_ISL_475608	B.1	20C
68878_S62	EPI_ISL_475609	B.1.43	20C
68887_S60	EPI_ISL_475611	B.1	20C
69048_S127	EPI_ISL_475612	A.3	19B
70047_S183	EPI_ISL_475613	A	19B
70748_S159	EPI_ISL_475614	B.1	20C
71234_S112	EPI_ISL_475615	B.1	20C
71240_S158	EPI_ISL_475616	B.1	20C
71257_S18	EPI_ISL_475617	B.1.43	20C
71687_S21	EPI_ISL_475619	B.1	20C
7193_S131	EPI_ISL_475620	B.1	20A
72077_S102	EPI_ISL_475621	B.1	20C
72189_S94	EPI_ISL_475622	B.1.2	20C
7916_S149	EPI_ISL_475623	B.1	19A

© 2020 Zhang W et al. *JAMA Network Open.*

eTable 3. Accession Number for GISAID SARS-CoV-2 Genomes Collected From Washington State; New York, New York; and China Analyzed With Integration of CSMC Samples

EPI_ISL_417130	EPI_ISL_417175	EPI_ISL_422510	EPI_ISL_422985	EPI_ISL_430122
EPI_ISL_417131	EPI_ISL_418029	EPI_ISL_422511	EPI_ISL_422986	EPI_ISL_430123
EPI_ISL_417132	EPI_ISL_418030	EPI_ISL_422512	EPI_ISL_422987	EPI_ISL_430124
EPI_ISL_417133	EPI_ISL_418031	EPI_ISL_422513	EPI_ISL_422988	EPI_ISL_430125
EPI_ISL_417134	EPI_ISL_418032	EPI_ISL_422514	EPI_ISL_422989	EPI_ISL_430126
EPI_ISL_417135	EPI_ISL_418033	EPI_ISL_422515	EPI_ISL_422990	EPI_ISL_430127
EPI_ISL_417136	EPI_ISL_418034	EPI_ISL_422516	EPI_ISL_422991	EPI_ISL_430128
EPI_ISL_417137	EPI_ISL_418037	EPI_ISL_422517	EPI_ISL_422992	EPI_ISL_430129
EPI_ISL_417138	EPI_ISL_418038	EPI_ISL_422518	EPI_ISL_422993	EPI_ISL_430130
EPI_ISL_417139	EPI_ISL_418052	EPI_ISL_422519	EPI_ISL_422994	EPI_ISL_430131
EPI_ISL_417140	EPI_ISL_418053	EPI_ISL_422520	EPI_ISL_422995	EPI_ISL_430132
EPI_ISL_417141	EPI_ISL_418054	EPI_ISL_422521	EPI_ISL_422996	EPI_ISL_430133
EPI_ISL_417142	EPI_ISL_418055	EPI_ISL_422522	EPI_ISL_422997	EPI_ISL_430134
EPI_ISL_417143	EPI_ISL_420300	EPI_ISL_422523	EPI_ISL_422998	EPI_ISL_430160
EPI_ISL_417144	EPI_ISL_420301	EPI_ISL_422524	EPI_ISL_422999	EPI_ISL_430161
EPI_ISL_417145	EPI_ISL_420302	EPI_ISL_422525	EPI_ISL_426080	EPI_ISL_430162
EPI_ISL_417146	EPI_ISL_420307	EPI_ISL_422526	EPI_ISL_426081	EPI_ISL_430163
EPI_ISL_417147	EPI_ISL_420308	EPI_ISL_422527	EPI_ISL_426082	EPI_ISL_430164
EPI_ISL_417148	EPI_ISL_420309	EPI_ISL_422528	EPI_ISL_426083	EPI_ISL_430165
EPI_ISL_417149	EPI_ISL_420793	EPI_ISL_422529	EPI_ISL_426084	EPI_ISL_430166
EPI_ISL_417150	EPI_ISL_421621	EPI_ISL_422530	EPI_ISL_426085	EPI_ISL_430167
EPI_ISL_417151	EPI_ISL_421622	EPI_ISL_422531	EPI_ISL_426086	EPI_ISL_430168
EPI_ISL_417152	EPI_ISL_421623	EPI_ISL_422532	EPI_ISL_426087	EPI_ISL_430169
EPI_ISL_417153	EPI_ISL_421624	EPI_ISL_422533	EPI_ISL_426088	EPI_ISL_430170
EPI_ISL_417154	EPI_ISL_421625	EPI_ISL_422550	EPI_ISL_426089	EPI_ISL_430171
EPI_ISL_417155	EPI_ISL_421626	EPI_ISL_422552	EPI_ISL_426090	EPI_ISL_430172
EPI_ISL_417156	EPI_ISL_421627	EPI_ISL_422553	EPI_ISL_426091	EPI_ISL_430173
EPI_ISL_417157	EPI_ISL_421628	EPI_ISL_422554	EPI_ISL_426092	EPI_ISL_430174
EPI_ISL_417158	EPI_ISL_421629	EPI_ISL_422555	EPI_ISL_426093	EPI_ISL_430175
EPI_ISL_417159	EPI_ISL_421630	EPI_ISL_422963	EPI_ISL_426094	EPI_ISL_430176
EPI_ISL_417160	EPI_ISL_421631	EPI_ISL_422964	EPI_ISL_426095	EPI_ISL_430177
EPI_ISL_417161	EPI_ISL_421632	EPI_ISL_422965	EPI_ISL_426096	EPI_ISL_430178
EPI_ISL_417162	EPI_ISL_421633	EPI_ISL_422966	EPI_ISL_426097	EPI_ISL_444732
EPI_ISL_417163	EPI_ISL_421634	EPI_ISL_422967	EPI_ISL_426098	EPI_ISL_444733
EPI_ISL_417164	EPI_ISL_421635	EPI_ISL_422969	EPI_ISL_426099	EPI_ISL_444734
EPI_ISL_417165	EPI_ISL_422500	EPI_ISL_422970	EPI_ISL_429597	EPI_ISL_444735
EPI_ISL_417166	EPI_ISL_422501	EPI_ISL_422971	EPI_ISL_429598	EPI_ISL_444736
EPI_ISL_417167	EPI_ISL_422502	EPI_ISL_422972	EPI_ISL_430113	EPI_ISL_444737
EPI_ISL_417168	EPI_ISL_422503	EPI_ISL_422973	EPI_ISL_430114	EPI_ISL_444738
EPI_ISL_417169	EPI_ISL_422504	EPI_ISL_422974	EPI_ISL_430115	EPI_ISL_444739
EPI_ISL_417170	EPI_ISL_422505	EPI_ISL_422975	EPI_ISL_430116	EPI_ISL_444740
EPI_ISL_417171	EPI_ISL_422506	EPI_ISL_422976	EPI_ISL_430117	EPI_ISL_444741
EPI_ISL_417172	EPI_ISL_422507	EPI_ISL_422977	EPI_ISL_430118	EPI_ISL_444743
EPI_ISL_417173	EPI_ISL_422508	EPI_ISL_422981	EPI_ISL_430119	EPI_ISL_444745
EPI_ISL_417174	EPI_ISL_422509	EPI_ISL_422984	EPI_ISL_430121	EPI_ISL_444746

© 2020 Zhang W et al. *JAMA Network Open.*

EPI_ISL_444747	EPI_ISL_416699	EPI_ISL_418880	EPI_ISL_426031	EPI_ISL_444711
EPI_ISL_444748	EPI_ISL_417100	EPI_ISL_418881	EPI_ISL_426032	EPI_ISL_444712
EPI_ISL_444749	EPI_ISL_417101	EPI_ISL_418882	EPI_ISL_426033	EPI_ISL_444713
EPI_ISL_444750	EPI_ISL_417102	EPI_ISL_418883	EPI_ISL_426034	EPI_ISL_444714
EPI_ISL_444751	EPI_ISL_417103	EPI_ISL_418884	EPI_ISL_426035	EPI_ISL_444715
EPI_ISL_444752	EPI_ISL_417104	EPI_ISL_418885	EPI_ISL_426036	EPI_ISL_444716
EPI_ISL_444753	EPI_ISL_417105	EPI_ISL_418886	EPI_ISL_426037	EPI_ISL_444717
EPI_ISL_444754	EPI_ISL_417106	EPI_ISL_418887	EPI_ISL_426038	EPI_ISL_444718
EPI_ISL_444756	EPI_ISL_417107	EPI_ISL_418888	EPI_ISL_426039	EPI_ISL_444719
EPI_ISL_444757	EPI_ISL_417108	EPI_ISL_418889	EPI_ISL_426040	EPI_ISL_444720
EPI_ISL_444758	EPI_ISL_417109	EPI_ISL_418890	EPI_ISL_426041	EPI_ISL_444721
EPI_ISL_444759	EPI_ISL_417110	EPI_ISL_418891	EPI_ISL_426042	EPI_ISL_444722
EPI_ISL_444760	EPI_ISL_417111	EPI_ISL_418892	EPI_ISL_426043	EPI_ISL_444723
EPI_ISL_444761	EPI_ISL_417112	EPI_ISL_418897	EPI_ISL_426044	EPI_ISL_444724
EPI_ISL_444762	EPI_ISL_417113	EPI_ISL_418899	EPI_ISL_426045	EPI_ISL_444725
EPI_ISL_444763	EPI_ISL_417114	EPI_ISL_421610	EPI_ISL_426046	EPI_ISL_444726
EPI_ISL_444764	EPI_ISL_417115	EPI_ISL_421611	EPI_ISL_426047	EPI_ISL_444727
EPI_ISL_444765	EPI_ISL_417116	EPI_ISL_421612	EPI_ISL_426048	EPI_ISL_444728
EPI_ISL_444766	EPI_ISL_417117	EPI_ISL_421613	EPI_ISL_426049	EPI_ISL_444729
EPI_ISL_444767	EPI_ISL_417118	EPI_ISL_421614	EPI_ISL_426079	EPI_ISL_444730
EPI_ISL_444768	EPI_ISL_417120	EPI_ISL_421615	EPI_ISL_430135	EPI_ISL_444731
EPI_ISL_444769	EPI_ISL_417121	EPI_ISL_421616	EPI_ISL_430136	EPI_ISL_444776
EPI_ISL_444770	EPI_ISL_417122	EPI_ISL_421617	EPI_ISL_430137	EPI_ISL_444777
EPI_ISL_444771	EPI_ISL_417123	EPI_ISL_421618	EPI_ISL_430139	EPI_ISL_444783
EPI_ISL_444772	EPI_ISL_417124	EPI_ISL_421620	EPI_ISL_430140	EPI_ISL_444784
EPI_ISL_444773	EPI_ISL_417125	EPI_ISL_422534	EPI_ISL_430141	EPI_ISL_444787
EPI_ISL_444774	EPI_ISL_417126	EPI_ISL_422535	EPI_ISL_430142	EPI_ISL_444788
EPI_ISL_444775	EPI_ISL_417127	EPI_ISL_422536	EPI_ISL_430143	EPI_ISL_444789
EPI_ISL_444781	EPI_ISL_417128	EPI_ISL_422537	EPI_ISL_430144	EPI_ISL_444790
EPI_ISL_444782	EPI_ISL_417129	EPI_ISL_422538	EPI_ISL_430146	EPI_ISL_444791
EPI_ISL_444786	EPI_ISL_418040	EPI_ISL_422539	EPI_ISL_430147	EPI_ISL_450100
EPI_ISL_413601	EPI_ISL_418046	EPI_ISL_422540	EPI_ISL_430148	EPI_ISL_450101
EPI_ISL_416680	EPI_ISL_418047	EPI_ISL_422541	EPI_ISL_430149	EPI_ISL_450102
EPI_ISL_416681	EPI_ISL_418048	EPI_ISL_422542	EPI_ISL_430150	EPI_ISL_450103
EPI_ISL_416683	EPI_ISL_418050	EPI_ISL_422543	EPI_ISL_430151	EPI_ISL_450104
EPI_ISL_416684	EPI_ISL_418866	EPI_ISL_422544	EPI_ISL_430152	EPI_ISL_450105
EPI_ISL_416685	EPI_ISL_418867	EPI_ISL_422545	EPI_ISL_430153	EPI_ISL_450106
EPI_ISL_416686	EPI_ISL_418868	EPI_ISL_422546	EPI_ISL_430154	EPI_ISL_450107
EPI_ISL_416687	EPI_ISL_418869	EPI_ISL_422547	EPI_ISL_430155	EPI_ISL_450108
EPI_ISL_416688	EPI_ISL_418870	EPI_ISL_422548	EPI_ISL_430156	EPI_ISL_450109
EPI_ISL_416689	EPI_ISL_418872	EPI_ISL_422549	EPI_ISL_430974	EPI_ISL_450110
EPI_ISL_416690	EPI_ISL_418873	EPI_ISL_422551	EPI_ISL_430975	EPI_ISL_450111
EPI_ISL_416691	EPI_ISL_418874	EPI_ISL_426025	EPI_ISL_430976	EPI_ISL_450112
EPI_ISL_416692	EPI_ISL_418875	EPI_ISL_426026	EPI_ISL_430977	EPI_ISL_450113
EPI_ISL_416693	EPI_ISL_418876	EPI_ISL_426027	EPI_ISL_430978	EPI_ISL_450114
EPI_ISL_416694	EPI_ISL_418877	EPI_ISL_426028	EPI_ISL_430979	EPI_ISL_450115
EPI_ISL_416695	EPI_ISL_418878	EPI_ISL_426029	EPI_ISL_430980	EPI_ISL_450116
EPI_ISL_416698	EPI_ISL_418879	EPI_ISL_426030	EPI_ISL_444710	EPI_ISL_450117

© 2020 Zhang W et al. *JAMA Network Open.*

EPI_ISL_450118	EPI_ISL_416635	EPI_ISL_421604	EPI_ISL_424262	EPI_ISL_426451
EPI_ISL_450119	EPI_ISL_416636	EPI_ISL_421605	EPI_ISL_424263	EPI_ISL_426452
EPI_ISL_450120	EPI_ISL_416637	EPI_ISL_421606	EPI_ISL_424264	EPI_ISL_426453
EPI_ISL_450121	EPI_ISL_416638	EPI_ISL_421607	EPI_ISL_424269	EPI_ISL_430900
EPI_ISL_450122	EPI_ISL_416639	EPI_ISL_421608	EPI_ISL_424270	EPI_ISL_430901
EPI_ISL_450123	EPI_ISL_416640	EPI_ISL_421609	EPI_ISL_424271	EPI_ISL_430902
EPI_ISL_450124	EPI_ISL_416641	EPI_ISL_424217	EPI_ISL_424272	EPI_ISL_430904
EPI_ISL_450125	EPI_ISL_416642	EPI_ISL_424218	EPI_ISL_424273	EPI_ISL_430905
EPI_ISL_450126	EPI_ISL_416643	EPI_ISL_424219	EPI_ISL_424274	EPI_ISL_430906
EPI_ISL_450127	EPI_ISL_416644	EPI_ISL_424220	EPI_ISL_424275	EPI_ISL_430911
EPI_ISL_450128	EPI_ISL_416645	EPI_ISL_424221	EPI_ISL_424276	EPI_ISL_430912
EPI_ISL_450129	EPI_ISL_416646	EPI_ISL_424222	EPI_ISL_424277	EPI_ISL_430913
EPI_ISL_450130	EPI_ISL_416647	EPI_ISL_424223	EPI_ISL_424278	EPI_ISL_430914
EPI_ISL_450131	EPI_ISL_416648	EPI_ISL_424224	EPI_ISL_424279	EPI_ISL_430915
EPI_ISL_450132	EPI_ISL_416649	EPI_ISL_424225	EPI_ISL_424280	EPI_ISL_430918
EPI_ISL_450133	EPI_ISL_416652	EPI_ISL_424226	EPI_ISL_424281	EPI_ISL_430920
EPI_ISL_450134	EPI_ISL_416653	EPI_ISL_424227	EPI_ISL_424282	EPI_ISL_430928
EPI_ISL_450135	EPI_ISL_416654	EPI_ISL_424228	EPI_ISL_424283	EPI_ISL_430929
EPI_ISL_450136	EPI_ISL_416655	EPI_ISL_424229	EPI_ISL_424284	EPI_ISL_430930
EPI_ISL_450137	EPI_ISL_416656	EPI_ISL_424230	EPI_ISL_424287	EPI_ISL_430931
EPI_ISL_450139	EPI_ISL_416657	EPI_ISL_424231	EPI_ISL_424288	EPI_ISL_430932
EPI_ISL_450144	EPI_ISL_416658	EPI_ISL_424232	EPI_ISL_424289	EPI_ISL_430933
EPI_ISL_450146	EPI_ISL_416659	EPI_ISL_424233	EPI_ISL_424290	EPI_ISL_430934
EPI_ISL_450147	EPI_ISL_416661	EPI_ISL_424234	EPI_ISL_424291	EPI_ISL_430935
EPI_ISL_450148	EPI_ISL_416662	EPI_ISL_424235	EPI_ISL_424292	EPI_ISL_430936
EPI_ISL_450149	EPI_ISL_416663	EPI_ISL_424236	EPI_ISL_424293	EPI_ISL_430937
EPI_ISL_450150	EPI_ISL_416664	EPI_ISL_424237	EPI_ISL_424294	EPI_ISL_430938
EPI_ISL_450151	EPI_ISL_416665	EPI_ISL_424238	EPI_ISL_424295	EPI_ISL_430939
EPI_ISL_450152	EPI_ISL_416666	EPI_ISL_424239	EPI_ISL_424296	EPI_ISL_430941
EPI_ISL_450153	EPI_ISL_416667	EPI_ISL_424240	EPI_ISL_424297	EPI_ISL_430943
EPI_ISL_450154	EPI_ISL_416668	EPI_ISL_424241	EPI_ISL_424298	EPI_ISL_430951
EPI_ISL_450155	EPI_ISL_416669	EPI_ISL_424242	EPI_ISL_424299	EPI_ISL_430953
EPI_ISL_450156	EPI_ISL_416670	EPI_ISL_424243	EPI_ISL_426050	EPI_ISL_430954
EPI_ISL_450157	EPI_ISL_416671	EPI_ISL_424244	EPI_ISL_426436	EPI_ISL_430955
EPI_ISL_450158	EPI_ISL_416677	EPI_ISL_424245	EPI_ISL_426437	EPI_ISL_430956
EPI_ISL_450159	EPI_ISL_416678	EPI_ISL_424246	EPI_ISL_426438	EPI_ISL_430957
EPI_ISL_450160	EPI_ISL_416679	EPI_ISL_424247	EPI_ISL_426439	EPI_ISL_430958
EPI_ISL_450161	EPI_ISL_417119	EPI_ISL_424250	EPI_ISL_426440	EPI_ISL_430959
EPI_ISL_450162	EPI_ISL_419700	EPI_ISL_424251	EPI_ISL_426441	EPI_ISL_430964
EPI_ISL_450163	EPI_ISL_419701	EPI_ISL_424252	EPI_ISL_426442	EPI_ISL_430966
EPI_ISL_450164	EPI_ISL_419702	EPI_ISL_424253	EPI_ISL_426443	EPI_ISL_430967
EPI_ISL_450165	EPI_ISL_419703	EPI_ISL_424254	EPI_ISL_426444	EPI_ISL_430968
EPI_ISL_450166	EPI_ISL_419704	EPI_ISL_424256	EPI_ISL_426445	EPI_ISL_430969
EPI_ISL_450167	EPI_ISL_419705	EPI_ISL_424257	EPI_ISL_426446	EPI_ISL_434063
EPI_ISL_450168	EPI_ISL_421600	EPI_ISL_424258	EPI_ISL_426447	EPI_ISL_434064
EPI_ISL_450169	EPI_ISL_421601	EPI_ISL_424259	EPI_ISL_426448	EPI_ISL_434065
EPI_ISL_450170	EPI_ISL_421602	EPI_ISL_424260	EPI_ISL_426449	EPI_ISL_434066
EPI_ISL_414476	EPI_ISL_421603	EPI_ISL_424261	EPI_ISL_426450	EPI_ISL_434067

© 2020 Zhang W et al. *JAMA Network Open.*

EPI_ISL_434068	EPI_ISL_449934	EPI_ISL_422491	EPI_ISL_430222	EPI_ISL_430271
EPI_ISL_434069	EPI_ISL_449990	EPI_ISL_422492	EPI_ISL_430223	EPI_ISL_430272
EPI_ISL_434070	EPI_ISL_449991	EPI_ISL_422493	EPI_ISL_430224	EPI_ISL_430273
EPI_ISL_434071	EPI_ISL_449992	EPI_ISL_422494	EPI_ISL_430225	EPI_ISL_430274
EPI_ISL_434072	EPI_ISL_449993	EPI_ISL_422495	EPI_ISL_430226	EPI_ISL_430275
EPI_ISL_434073	EPI_ISL_449994	EPI_ISL_422496	EPI_ISL_430227	EPI_ISL_430276
EPI_ISL_434074	EPI_ISL_449995	EPI_ISL_422497	EPI_ISL_430228	EPI_ISL_430277
EPI_ISL_434075	EPI_ISL_449996	EPI_ISL_422498	EPI_ISL_430229	EPI_ISL_430278
EPI_ISL_434076	EPI_ISL_449997	EPI_ISL_422499	EPI_ISL_430230	EPI_ISL_430279
EPI_ISL_434077	EPI_ISL_449998	EPI_ISL_424200	EPI_ISL_430231	EPI_ISL_430280
EPI_ISL_434078	EPI_ISL_449999	EPI_ISL_424201	EPI_ISL_430232	EPI_ISL_430281
EPI_ISL_434079	EPI_ISL_450140	EPI_ISL_424202	EPI_ISL_430233	EPI_ISL_430282
EPI_ISL_434080	EPI_ISL_450141	EPI_ISL_424203	EPI_ISL_430234	EPI_ISL_430283
EPI_ISL_434081	EPI_ISL_450142	EPI_ISL_424204	EPI_ISL_430235	EPI_ISL_430284
EPI_ISL_434082	EPI_ISL_450143	EPI_ISL_424205	EPI_ISL_430236	EPI_ISL_430285
EPI_ISL_434083	EPI_ISL_413560	EPI_ISL_424206	EPI_ISL_430237	EPI_ISL_430286
EPI_ISL_434084	EPI_ISL_413562	EPI_ISL_424207	EPI_ISL_430238	EPI_ISL_430287
EPI_ISL_434085	EPI_ISL_413563	EPI_ISL_424208	EPI_ISL_430239	EPI_ISL_430288
EPI_ISL_434086	EPI_ISL_417090	EPI_ISL_424209	EPI_ISL_430240	EPI_ISL_430289
EPI_ISL_434087	EPI_ISL_417091	EPI_ISL_424210	EPI_ISL_430241	EPI_ISL_430290
EPI_ISL_434088	EPI_ISL_417092	EPI_ISL_424211	EPI_ISL_430242	EPI_ISL_430291
EPI_ISL_434089	EPI_ISL_417093	EPI_ISL_424212	EPI_ISL_430243	EPI_ISL_430292
EPI_ISL_434090	EPI_ISL_417094	EPI_ISL_424213	EPI_ISL_430244	EPI_ISL_430293
EPI_ISL_434091	EPI_ISL_417095	EPI_ISL_424214	EPI_ISL_430245	EPI_ISL_430294
EPI_ISL_434092	EPI_ISL_417096	EPI_ISL_424215	EPI_ISL_430246	EPI_ISL_430295
EPI_ISL_434093	EPI_ISL_417097	EPI_ISL_424216	EPI_ISL_430247	EPI_ISL_435500
EPI_ISL_434094	EPI_ISL_417098	EPI_ISL_427470	EPI_ISL_430248	EPI_ISL_435501
EPI_ISL_434095	EPI_ISL_417099	EPI_ISL_427471	EPI_ISL_430249	EPI_ISL_435502
EPI_ISL_434096	EPI_ISL_420293	EPI_ISL_427472	EPI_ISL_430250	EPI_ISL_438180
EPI_ISL_434097	EPI_ISL_420296	EPI_ISL_427473	EPI_ISL_430251	EPI_ISL_438181
EPI_ISL_434098	EPI_ISL_420297	EPI_ISL_427474	EPI_ISL_430252	EPI_ISL_438182
EPI_ISL_434099	EPI_ISL_420298	EPI_ISL_427475	EPI_ISL_430253	EPI_ISL_438183
EPI_ISL_444700	EPI_ISL_420299	EPI_ISL_427476	EPI_ISL_430254	EPI_ISL_438184
EPI_ISL_444701	EPI_ISL_421588	EPI_ISL_429650	EPI_ISL_430255	EPI_ISL_438185
EPI_ISL_444702	EPI_ISL_421589	EPI_ISL_429651	EPI_ISL_430256	EPI_ISL_438186
EPI_ISL_444703	EPI_ISL_421590	EPI_ISL_429652	EPI_ISL_430257	EPI_ISL_438187
EPI_ISL_444704	EPI_ISL_421591	EPI_ISL_429653	EPI_ISL_430258	EPI_ISL_438188
EPI_ISL_444705	EPI_ISL_421592	EPI_ISL_429655	EPI_ISL_430259	EPI_ISL_438189
EPI_ISL_444706	EPI_ISL_421593	EPI_ISL_430212	EPI_ISL_430261	EPI_ISL_438190
EPI_ISL_444707	EPI_ISL_421594	EPI_ISL_430213	EPI_ISL_430262	EPI_ISL_438191
EPI_ISL_444708	EPI_ISL_421595	EPI_ISL_430214	EPI_ISL_430263	EPI_ISL_438192
EPI_ISL_444709	EPI_ISL_421596	EPI_ISL_430215	EPI_ISL_430264	EPI_ISL_438193
EPI_ISL_449928	EPI_ISL_421597	EPI_ISL_430216	EPI_ISL_430265	EPI_ISL_438194
EPI_ISL_449929	EPI_ISL_421598	EPI_ISL_430217	EPI_ISL_430266	EPI_ISL_438195
EPI_ISL_449930	EPI_ISL_421599	EPI_ISL_430218	EPI_ISL_430267	EPI_ISL_438196
EPI_ISL_449931	EPI_ISL_422488	EPI_ISL_430219	EPI_ISL_430268	EPI_ISL_438197
EPI_ISL_449932	EPI_ISL_422489	EPI_ISL_430220	EPI_ISL_430269	EPI_ISL_438198
EPI_ISL_449933	EPI_ISL_422490	EPI_ISL_430221	EPI_ISL_430270	EPI_ISL_438199

© 2020 Zhang W et al. *JAMA Network Open.*

EPI_ISL_444699	EPI_ISL_449958	EPI_ISL_418904	EPI_ISL_421733	EPI_ISL_427479
EPI_ISL_449902	EPI_ISL_449959	EPI_ISL_418905	EPI_ISL_423007	EPI_ISL_427480
EPI_ISL_449903	EPI_ISL_449960	EPI_ISL_418906	EPI_ISL_423008	EPI_ISL_427481
EPI_ISL_449904	EPI_ISL_449961	EPI_ISL_418907	EPI_ISL_423009	EPI_ISL_427482
EPI_ISL_449905	EPI_ISL_449962	EPI_ISL_418908	EPI_ISL_423010	EPI_ISL_427483
EPI_ISL_449906	EPI_ISL_449963	EPI_ISL_418909	EPI_ISL_423011	EPI_ISL_427484
EPI_ISL_449907	EPI_ISL_449964	EPI_ISL_418910	EPI_ISL_423012	EPI_ISL_427485
EPI_ISL_449908	EPI_ISL_449965	EPI_ISL_418911	EPI_ISL_423013	EPI_ISL_427486
EPI_ISL_449909	EPI_ISL_449966	EPI_ISL_418912	EPI_ISL_423014	EPI_ISL_427487
EPI_ISL_449910	EPI_ISL_449967	EPI_ISL_418913	EPI_ISL_423015	EPI_ISL_427488
EPI_ISL_449911	EPI_ISL_449968	EPI_ISL_418914	EPI_ISL_423016	EPI_ISL_427489
EPI_ISL_449912	EPI_ISL_449969	EPI_ISL_418928	EPI_ISL_423017	EPI_ISL_427490
EPI_ISL_449913	EPI_ISL_449970	EPI_ISL_418929	EPI_ISL_423018	EPI_ISL_427491
EPI_ISL_449914	EPI_ISL_449971	EPI_ISL_418930	EPI_ISL_423019	EPI_ISL_427492
EPI_ISL_449915	EPI_ISL_449972	EPI_ISL_418931	EPI_ISL_423020	EPI_ISL_427493
EPI_ISL_449916	EPI_ISL_449973	EPI_ISL_418932	EPI_ISL_423021	EPI_ISL_427494
EPI_ISL_449917	EPI_ISL_449974	EPI_ISL_418933	EPI_ISL_423022	EPI_ISL_427495
EPI_ISL_449918	EPI_ISL_449975	EPI_ISL_418934	EPI_ISL_423024	EPI_ISL_427496
EPI_ISL_449919	EPI_ISL_449977	EPI_ISL_418935	EPI_ISL_423025	EPI_ISL_427497
EPI_ISL_449920	EPI_ISL_449978	EPI_ISL_418936	EPI_ISL_423026	EPI_ISL_427498
EPI_ISL_449921	EPI_ISL_449979	EPI_ISL_418980	EPI_ISL_423027	EPI_ISL_427499
EPI_ISL_449922	EPI_ISL_449980	EPI_ISL_421704	EPI_ISL_423028	EPI_ISL_428780
EPI_ISL_449923	EPI_ISL_449981	EPI_ISL_421705	EPI_ISL_424338	EPI_ISL_428781
EPI_ISL_449924	EPI_ISL_449982	EPI_ISL_421706	EPI_ISL_424339	EPI_ISL_428782
EPI_ISL_449925	EPI_ISL_449983	EPI_ISL_421707	EPI_ISL_424340	EPI_ISL_428783
EPI_ISL_449926	EPI_ISL_449984	EPI_ISL_421708	EPI_ISL_424341	EPI_ISL_428784
EPI_ISL_449927	EPI_ISL_449985	EPI_ISL_421709	EPI_ISL_425224	EPI_ISL_428785
EPI_ISL_449935	EPI_ISL_449986	EPI_ISL_421710	EPI_ISL_426120	EPI_ISL_428786
EPI_ISL_449936	EPI_ISL_449987	EPI_ISL_421711	EPI_ISL_426121	EPI_ISL_428787
EPI_ISL_449937	EPI_ISL_449988	EPI_ISL_421712	EPI_ISL_426122	EPI_ISL_428788
EPI_ISL_449938	EPI_ISL_449989	EPI_ISL_421713	EPI_ISL_426123	EPI_ISL_428789
EPI_ISL_449939	EPI_ISL_416715	EPI_ISL_421714	EPI_ISL_426124	EPI_ISL_428790
EPI_ISL_449940	EPI_ISL_416716	EPI_ISL_421715	EPI_ISL_426125	EPI_ISL_428791
EPI_ISL_449941	EPI_ISL_416717	EPI_ISL_421716	EPI_ISL_426126	EPI_ISL_428792
EPI_ISL_449943	EPI_ISL_416718	EPI_ISL_421717	EPI_ISL_426127	EPI_ISL_428793
EPI_ISL_449944	EPI_ISL_416719	EPI_ISL_421719	EPI_ISL_426128	EPI_ISL_428794
EPI_ISL_449945	EPI_ISL_416720	EPI_ISL_421720	EPI_ISL_426129	EPI_ISL_428795
EPI_ISL_449946	EPI_ISL_416721	EPI_ISL_421721	EPI_ISL_426130	EPI_ISL_428796
EPI_ISL_449947	EPI_ISL_416722	EPI_ISL_421722	EPI_ISL_426131	EPI_ISL_428797
EPI_ISL_449948	EPI_ISL_416723	EPI_ISL_421724	EPI_ISL_426132	EPI_ISL_428798
EPI_ISL_449949	EPI_ISL_416724	EPI_ISL_421725	EPI_ISL_426133	EPI_ISL_428799
EPI_ISL_449951	EPI_ISL_416725	EPI_ISL_421726	EPI_ISL_426134	EPI_ISL_429629
EPI_ISL_449952	EPI_ISL_416726	EPI_ISL_421727	EPI_ISL_426135	EPI_ISL_429630
EPI_ISL_449953	EPI_ISL_416727	EPI_ISL_421728	EPI_ISL_426136	EPI_ISL_429631
EPI_ISL_449954	EPI_ISL_416728	EPI_ISL_421729	EPI_ISL_426137	EPI_ISL_429632
EPI_ISL_449955	EPI_ISL_416729	EPI_ISL_421730	EPI_ISL_427469	EPI_ISL_429633
EPI_ISL_449956	EPI_ISL_418900	EPI_ISL_421731	EPI_ISL_427477	EPI_ISL_429634
EPI_ISL_449957	EPI_ISL_418903	EPI_ISL_421732	EPI_ISL_427478	EPI_ISL_429635

EPI_ISL_429636	EPI_ISL_435493	EPI_ISL_413650	EPI_ISL_418951	EPI_ISL_426107
EPI_ISL_429637	EPI_ISL_435494	EPI_ISL_413651	EPI_ISL_418952	EPI_ISL_426108
EPI_ISL_429638	EPI_ISL_435495	EPI_ISL_413652	EPI_ISL_418953	EPI_ISL_426109
EPI_ISL_429639	EPI_ISL_435496	EPI_ISL_413653	EPI_ISL_418954	EPI_ISL_426110
EPI_ISL_429644	EPI_ISL_435497	EPI_ISL_414591	EPI_ISL_418955	EPI_ISL_426111
EPI_ISL_429647	EPI_ISL_435498	EPI_ISL_414592	EPI_ISL_418968	EPI_ISL_426112
EPI_ISL_429648	EPI_ISL_435499	EPI_ISL_414593	EPI_ISL_418969	EPI_ISL_426113
EPI_ISL_429649	EPI_ISL_438140	EPI_ISL_414594	EPI_ISL_418970	EPI_ISL_426114
EPI_ISL_429654	EPI_ISL_438141	EPI_ISL_414595	EPI_ISL_418971	EPI_ISL_426115
EPI_ISL_430200	EPI_ISL_438142	EPI_ISL_414596	EPI_ISL_418972	EPI_ISL_426116
EPI_ISL_430201	EPI_ISL_438143	EPI_ISL_414597	EPI_ISL_418973	EPI_ISL_426117
EPI_ISL_430202	EPI_ISL_438144	EPI_ISL_418062	EPI_ISL_418974	EPI_ISL_426118
EPI_ISL_430203	EPI_ISL_438145	EPI_ISL_418063	EPI_ISL_418975	EPI_ISL_426119
EPI_ISL_430204	EPI_ISL_438146	EPI_ISL_418064	EPI_ISL_418976	EPI_ISL_428757
EPI_ISL_430205	EPI_ISL_438147	EPI_ISL_418065	EPI_ISL_418977	EPI_ISL_428758
EPI_ISL_430206	EPI_ISL_438148	EPI_ISL_418066	EPI_ISL_418978	EPI_ISL_428759
EPI_ISL_430207	EPI_ISL_438149	EPI_ISL_418067	EPI_ISL_418979	EPI_ISL_428760
EPI_ISL_430208	EPI_ISL_438150	EPI_ISL_418068	EPI_ISL_422556	EPI_ISL_428761
EPI_ISL_430209	EPI_ISL_438151	EPI_ISL_418069	EPI_ISL_422557	EPI_ISL_428762
EPI_ISL_430210	EPI_ISL_438152	EPI_ISL_418070	EPI_ISL_422558	EPI_ISL_428763
EPI_ISL_430211	EPI_ISL_438153	EPI_ISL_418071	EPI_ISL_422559	EPI_ISL_428764
EPI_ISL_434139	EPI_ISL_438154	EPI_ISL_418072	EPI_ISL_422560	EPI_ISL_428765
EPI_ISL_434146	EPI_ISL_438155	EPI_ISL_418073	EPI_ISL_422561	EPI_ISL_428766
EPI_ISL_434147	EPI_ISL_438156	EPI_ISL_418074	EPI_ISL_422562	EPI_ISL_428767
EPI_ISL_434160	EPI_ISL_438157	EPI_ISL_418075	EPI_ISL_423029	EPI_ISL_428768
EPI_ISL_434161	EPI_ISL_438158	EPI_ISL_418076	EPI_ISL_423030	EPI_ISL_428769
EPI_ISL_434162	EPI_ISL_438159	EPI_ISL_418077	EPI_ISL_423031	EPI_ISL_428770
EPI_ISL_434163	EPI_ISL_438160	EPI_ISL_418078	EPI_ISL_423032	EPI_ISL_428771
EPI_ISL_434164	EPI_ISL_438161	EPI_ISL_418079	EPI_ISL_423033	EPI_ISL_428772
EPI_ISL_434165	EPI_ISL_438162	EPI_ISL_418080	EPI_ISL_424300	EPI_ISL_428773
EPI_ISL_434166	EPI_ISL_438163	EPI_ISL_418081	EPI_ISL_424301	EPI_ISL_428774
EPI_ISL_434167	EPI_ISL_438164	EPI_ISL_418082	EPI_ISL_424302	EPI_ISL_428775
EPI_ISL_434168	EPI_ISL_438165	EPI_ISL_418922	EPI_ISL_424304	EPI_ISL_428776
EPI_ISL_434169	EPI_ISL_438166	EPI_ISL_418923	EPI_ISL_424305	EPI_ISL_428777
EPI_ISL_434170	EPI_ISL_438167	EPI_ISL_418924	EPI_ISL_424306	EPI_ISL_428778
EPI_ISL_434171	EPI_ISL_438168	EPI_ISL_418926	EPI_ISL_424309	EPI_ISL_428779
EPI_ISL_434172	EPI_ISL_438169	EPI_ISL_418927	EPI_ISL_424310	EPI_ISL_429600
EPI_ISL_434173	EPI_ISL_438170	EPI_ISL_418937	EPI_ISL_424311	EPI_ISL_429602
EPI_ISL_434174	EPI_ISL_438171	EPI_ISL_418938	EPI_ISL_424312	EPI_ISL_429603
EPI_ISL_434175	EPI_ISL_438172	EPI_ISL_418939	EPI_ISL_424313	EPI_ISL_429604
EPI_ISL_434176	EPI_ISL_438173	EPI_ISL_418940	EPI_ISL_424314	EPI_ISL_429605
EPI_ISL_434177	EPI_ISL_438174	EPI_ISL_418942	EPI_ISL_426100	EPI_ISL_429606
EPI_ISL_434178	EPI_ISL_438175	EPI_ISL_418943	EPI_ISL_426101	EPI_ISL_429607
EPI_ISL_434179	EPI_ISL_438176	EPI_ISL_418944	EPI_ISL_426102	EPI_ISL_429608
EPI_ISL_435478	EPI_ISL_438177	EPI_ISL_418945	EPI_ISL_426103	EPI_ISL_429609
EPI_ISL_435490	EPI_ISL_438178	EPI_ISL_418948	EPI_ISL_426104	EPI_ISL_429611
EPI_ISL_435491	EPI_ISL_438179	EPI_ISL_418949	EPI_ISL_426105	EPI_ISL_429612
EPI_ISL_435492	EPI_ISL_413649	EPI_ISL_418950	EPI_ISL_426106	EPI_ISL_429613

© 2020 Zhang W et al. *JAMA Network Open.*

EPI_ISL_429614	EPI_ISL_434132	EPI_ISL_435482	EPI_ISL_420574	EPI_ISL_421431
EPI_ISL_429615	EPI_ISL_434135	EPI_ISL_435484	EPI_ISL_420575	EPI_ISL_421432
EPI_ISL_429616	EPI_ISL_434136	EPI_ISL_435485	EPI_ISL_420576	EPI_ISL_421433
EPI_ISL_429617	EPI_ISL_434137	EPI_ISL_435486	EPI_ISL_420577	EPI_ISL_421434
EPI_ISL_429618	EPI_ISL_434138	EPI_ISL_435487	EPI_ISL_420578	EPI_ISL_421435
EPI_ISL_429619	EPI_ISL_434140	EPI_ISL_435489	EPI_ISL_420579	EPI_ISL_423000
EPI_ISL_429620	EPI_ISL_434141	EPI_ISL_436733	EPI_ISL_420580	EPI_ISL_423001
EPI_ISL_429621	EPI_ISL_434142	EPI_ISL_436734	EPI_ISL_420581	EPI_ISL_423002
EPI_ISL_429622	EPI_ISL_434143	EPI_ISL_436735	EPI_ISL_420582	EPI_ISL_423003
EPI_ISL_429623	EPI_ISL_434144	EPI_ISL_436736	EPI_ISL_420584	EPI_ISL_423004
EPI_ISL_429624	EPI_ISL_434145	EPI_ISL_436737	EPI_ISL_420585	EPI_ISL_423005
EPI_ISL_429625	EPI_ISL_434148	EPI_ISL_436738	EPI_ISL_420586	EPI_ISL_423006
EPI_ISL_429626	EPI_ISL_434149	EPI_ISL_436740	EPI_ISL_420587	EPI_ISL_424316
EPI_ISL_429627	EPI_ISL_434150	EPI_ISL_436741	EPI_ISL_420588	EPI_ISL_424317
EPI_ISL_430179	EPI_ISL_434151	EPI_ISL_436743	EPI_ISL_420589	EPI_ISL_424323
EPI_ISL_430180	EPI_ISL_434152	EPI_ISL_412970	EPI_ISL_420590	EPI_ISL_424324
EPI_ISL_430181	EPI_ISL_434153	EPI_ISL_416700	EPI_ISL_420591	EPI_ISL_424325
EPI_ISL_430182	EPI_ISL_434154	EPI_ISL_416701	EPI_ISL_421400	EPI_ISL_424326
EPI_ISL_430183	EPI_ISL_434155	EPI_ISL_416702	EPI_ISL_421401	EPI_ISL_424327
EPI_ISL_430184	EPI_ISL_434156	EPI_ISL_416703	EPI_ISL_421402	EPI_ISL_424328
EPI_ISL_430185	EPI_ISL_434157	EPI_ISL_416704	EPI_ISL_421403	EPI_ISL_424329
EPI_ISL_430186	EPI_ISL_434158	EPI_ISL_416705	EPI_ISL_421404	EPI_ISL_424330
EPI_ISL_430187	EPI_ISL_434159	EPI_ISL_416706	EPI_ISL_421405	EPI_ISL_424331
EPI_ISL_430188	EPI_ISL_434180	EPI_ISL_416707	EPI_ISL_421406	EPI_ISL_424332
EPI_ISL_430189	EPI_ISL_434182	EPI_ISL_416708	EPI_ISL_421407	EPI_ISL_424333
EPI_ISL_430190	EPI_ISL_434183	EPI_ISL_416709	EPI_ISL_421408	EPI_ISL_424334
EPI_ISL_430191	EPI_ISL_434184	EPI_ISL_416710	EPI_ISL_421409	EPI_ISL_424335
EPI_ISL_430192	EPI_ISL_434185	EPI_ISL_416711	EPI_ISL_421410	EPI_ISL_424336
EPI_ISL_430193	EPI_ISL_434186	EPI_ISL_416712	EPI_ISL_421411	EPI_ISL_424337
EPI_ISL_430194	EPI_ISL_434187	EPI_ISL_416713	EPI_ISL_421412	EPI_ISL_424906
EPI_ISL_430196	EPI_ISL_434188	EPI_ISL_416714	EPI_ISL_421413	EPI_ISL_424929
EPI_ISL_430197	EPI_ISL_434189	EPI_ISL_417370	EPI_ISL_421414	EPI_ISL_424930
EPI_ISL_430198	EPI_ISL_434190	EPI_ISL_417371	EPI_ISL_421415	EPI_ISL_424931
EPI_ISL_430199	EPI_ISL_434191	EPI_ISL_417374	EPI_ISL_421416	EPI_ISL_424932
EPI_ISL_434117	EPI_ISL_434192	EPI_ISL_417375	EPI_ISL_421417	EPI_ISL_424933
EPI_ISL_434118	EPI_ISL_434193	EPI_ISL_417376	EPI_ISL_421418	EPI_ISL_424934
EPI_ISL_434119	EPI_ISL_434194	EPI_ISL_417377	EPI_ISL_421419	EPI_ISL_424935
EPI_ISL_434120	EPI_ISL_434195	EPI_ISL_417378	EPI_ISL_421420	EPI_ISL_424936
EPI_ISL_434121	EPI_ISL_434196	EPI_ISL_417379	EPI_ISL_421421	EPI_ISL_424937
EPI_ISL_434122	EPI_ISL_434197	EPI_ISL_417380	EPI_ISL_421422	EPI_ISL_424938
EPI_ISL_434123	EPI_ISL_434198	EPI_ISL_417381	EPI_ISL_421423	EPI_ISL_424939
EPI_ISL_434124	EPI_ISL_434199	EPI_ISL_417382	EPI_ISL_421424	EPI_ISL_424940
EPI_ISL_434125	EPI_ISL_435475	EPI_ISL_418254	EPI_ISL_421425	EPI_ISL_424941
EPI_ISL_434126	EPI_ISL_435476	EPI_ISL_419555	EPI_ISL_421426	EPI_ISL_424942
EPI_ISL_434127	EPI_ISL_435477	EPI_ISL_420570	EPI_ISL_421427	EPI_ISL_424943
EPI_ISL_434128	EPI_ISL_435479	EPI_ISL_420571	EPI_ISL_421428	EPI_ISL_424944
EPI_ISL_434129	EPI_ISL_435480	EPI_ISL_420572	EPI_ISL_421429	EPI_ISL_424945
EPI_ISL_434131	EPI_ISL_435481	EPI_ISL_420573	EPI_ISL_421430	EPI_ISL_424946

© 2020 Zhang W et al. *JAMA Network Open.*

EPI_ISL_424947	EPI_ISL_430338	EPI_ISL_430388	EPI_ISL_416446	EPI_ISL_418202
EPI_ISL_424948	EPI_ISL_430339	EPI_ISL_430389	EPI_ISL_416447	EPI_ISL_418203
EPI_ISL_424949	EPI_ISL_430340	EPI_ISL_430391	EPI_ISL_416448	EPI_ISL_418204
EPI_ISL_424950	EPI_ISL_430341	EPI_ISL_430392	EPI_ISL_416449	EPI_ISL_418205
EPI_ISL_424951	EPI_ISL_430342	EPI_ISL_430393	EPI_ISL_416450	EPI_ISL_419523
EPI_ISL_424952	EPI_ISL_430343	EPI_ISL_430395	EPI_ISL_416451	EPI_ISL_424954
EPI_ISL_424953	EPI_ISL_430344	EPI_ISL_430396	EPI_ISL_416452	EPI_ISL_424955
EPI_ISL_424959	EPI_ISL_430345	EPI_ISL_430397	EPI_ISL_416453	EPI_ISL_424956
EPI_ISL_424960	EPI_ISL_430346	EPI_ISL_430398	EPI_ISL_416454	EPI_ISL_424957
EPI_ISL_424961	EPI_ISL_430347	EPI_ISL_430399	EPI_ISL_416455	EPI_ISL_424958
EPI_ISL_424962	EPI_ISL_430348	EPI_ISL_434100	EPI_ISL_416456	EPI_ISL_426290
EPI_ISL_424963	EPI_ISL_430349	EPI_ISL_434101	EPI_ISL_416459	EPI_ISL_426291
EPI_ISL_424964	EPI_ISL_430350	EPI_ISL_434102	EPI_ISL_416460	EPI_ISL_426292
EPI_ISL_424965	EPI_ISL_430351	EPI_ISL_434103	EPI_ISL_416461	EPI_ISL_426293
EPI_ISL_424966	EPI_ISL_430352	EPI_ISL_434104	EPI_ISL_416462	EPI_ISL_426294
EPI_ISL_424967	EPI_ISL_430353	EPI_ISL_434105	EPI_ISL_416463	EPI_ISL_426295
EPI_ISL_424968	EPI_ISL_430354	EPI_ISL_434106	EPI_ISL_416464	EPI_ISL_426296
EPI_ISL_427174	EPI_ISL_430355	EPI_ISL_434107	EPI_ISL_416465	EPI_ISL_426297
EPI_ISL_427175	EPI_ISL_430357	EPI_ISL_434108	EPI_ISL_416466	EPI_ISL_426298
EPI_ISL_427176	EPI_ISL_430358	EPI_ISL_434109	EPI_ISL_417341	EPI_ISL_426299
EPI_ISL_427177	EPI_ISL_430359	EPI_ISL_434110	EPI_ISL_417342	EPI_ISL_427500
EPI_ISL_427178	EPI_ISL_430360	EPI_ISL_434111	EPI_ISL_417343	EPI_ISL_427501
EPI_ISL_427179	EPI_ISL_430361	EPI_ISL_434112	EPI_ISL_417344	EPI_ISL_427502
EPI_ISL_427180	EPI_ISL_430362	EPI_ISL_434113	EPI_ISL_417345	EPI_ISL_427503
EPI_ISL_427181	EPI_ISL_430363	EPI_ISL_434114	EPI_ISL_417346	EPI_ISL_427504
EPI_ISL_427182	EPI_ISL_430364	EPI_ISL_434115	EPI_ISL_417347	EPI_ISL_427505
EPI_ISL_427183	EPI_ISL_430365	EPI_ISL_434116	EPI_ISL_417348	EPI_ISL_427506
EPI_ISL_427184	EPI_ISL_430366	EPI_ISL_434130	EPI_ISL_417349	EPI_ISL_427507
EPI_ISL_427185	EPI_ISL_430367	EPI_ISL_434133	EPI_ISL_417350	EPI_ISL_427508
EPI_ISL_427186	EPI_ISL_430368	EPI_ISL_434134	EPI_ISL_417351	EPI_ISL_427509
EPI_ISL_427187	EPI_ISL_430369	EPI_ISL_444518	EPI_ISL_417352	EPI_ISL_427510
EPI_ISL_427188	EPI_ISL_430370	EPI_ISL_404895	EPI_ISL_417353	EPI_ISL_427511
EPI_ISL_427189	EPI_ISL_430372	EPI_ISL_415151	EPI_ISL_417354	EPI_ISL_427512
EPI_ISL_427190	EPI_ISL_430373	EPI_ISL_415591	EPI_ISL_417355	EPI_ISL_427513
EPI_ISL_427191	EPI_ISL_430374	EPI_ISL_415592	EPI_ISL_417356	EPI_ISL_427514
EPI_ISL_427192	EPI_ISL_430375	EPI_ISL_415593	EPI_ISL_417357	EPI_ISL_427515
EPI_ISL_427193	EPI_ISL_430376	EPI_ISL_415594	EPI_ISL_417358	EPI_ISL_427516
EPI_ISL_427194	EPI_ISL_430377	EPI_ISL_415595	EPI_ISL_417359	EPI_ISL_427517
EPI_ISL_427195	EPI_ISL_430378	EPI_ISL_415596	EPI_ISL_417360	EPI_ISL_427518
EPI_ISL_427196	EPI_ISL_430379	EPI_ISL_415597	EPI_ISL_417361	EPI_ISL_427519
EPI_ISL_427197	EPI_ISL_430380	EPI_ISL_415598	EPI_ISL_417363	EPI_ISL_427520
EPI_ISL_427198	EPI_ISL_430381	EPI_ISL_415599	EPI_ISL_417364	EPI_ISL_427521
EPI_ISL_427199	EPI_ISL_430382	EPI_ISL_416440	EPI_ISL_417365	EPI_ISL_427524
EPI_ISL_430333	EPI_ISL_430383	EPI_ISL_416441	EPI_ISL_417366	EPI_ISL_427525
EPI_ISL_430334	EPI_ISL_430384	EPI_ISL_416442	EPI_ISL_417367	EPI_ISL_427526
EPI_ISL_430335	EPI_ISL_430385	EPI_ISL_416443	EPI_ISL_417369	EPI_ISL_427527
EPI_ISL_430336	EPI_ISL_430386	EPI_ISL_416444	EPI_ISL_418200	EPI_ISL_427550
EPI_ISL_430337	EPI_ISL_430387	EPI_ISL_416445	EPI_ISL_418201	EPI_ISL_427551

© 2020 Zhang W et al. *JAMA Network Open.*

EPI_ISL_427552	EPI_ISL_430390	EPI_ISL_436050	EPI_ISL_414621	EPI_ISL_421370
EPI_ISL_427553	EPI_ISL_434260	EPI_ISL_436051	EPI_ISL_414622	EPI_ISL_421371
EPI_ISL_427554	EPI_ISL_434261	EPI_ISL_436052	EPI_ISL_414639	EPI_ISL_421372
EPI_ISL_427555	EPI_ISL_434262	EPI_ISL_436053	EPI_ISL_416433	EPI_ISL_421373
EPI_ISL_427556	EPI_ISL_434263	EPI_ISL_436054	EPI_ISL_416434	EPI_ISL_421374
EPI_ISL_427557	EPI_ISL_434264	EPI_ISL_436055	EPI_ISL_416435	EPI_ISL_421375
EPI_ISL_427558	EPI_ISL_434265	EPI_ISL_436056	EPI_ISL_416436	EPI_ISL_421376
EPI_ISL_427559	EPI_ISL_434266	EPI_ISL_436057	EPI_ISL_416437	EPI_ISL_421377
EPI_ISL_427570	EPI_ISL_434267	EPI_ISL_436058	EPI_ISL_416438	EPI_ISL_421378
EPI_ISL_427571	EPI_ISL_434268	EPI_ISL_436059	EPI_ISL_416439	EPI_ISL_421379
EPI_ISL_427572	EPI_ISL_434269	EPI_ISL_436060	EPI_ISL_416830	EPI_ISL_421380
EPI_ISL_427573	EPI_ISL_434270	EPI_ISL_436061	EPI_ISL_416831	EPI_ISL_421381
EPI_ISL_427574	EPI_ISL_434271	EPI_ISL_436062	EPI_ISL_416832	EPI_ISL_421382
EPI_ISL_427575	EPI_ISL_434272	EPI_ISL_436063	EPI_ISL_418190	EPI_ISL_421383
EPI_ISL_427576	EPI_ISL_434273	EPI_ISL_436064	EPI_ISL_418191	EPI_ISL_421384
EPI_ISL_427577	EPI_ISL_434274	EPI_ISL_436065	EPI_ISL_418192	EPI_ISL_421385
EPI_ISL_427578	EPI_ISL_434275	EPI_ISL_436066	EPI_ISL_418193	EPI_ISL_421386
EPI_ISL_427579	EPI_ISL_434276	EPI_ISL_436067	EPI_ISL_418194	EPI_ISL_421387
EPI_ISL_427580	EPI_ISL_434277	EPI_ISL_436068	EPI_ISL_418195	EPI_ISL_421388
EPI_ISL_427581	EPI_ISL_434278	EPI_ISL_436069	EPI_ISL_418196	EPI_ISL_421389
EPI_ISL_427582	EPI_ISL_434279	EPI_ISL_436070	EPI_ISL_418197	EPI_ISL_421390
EPI_ISL_427583	EPI_ISL_434280	EPI_ISL_436071	EPI_ISL_418198	EPI_ISL_421391
EPI_ISL_427584	EPI_ISL_434281	EPI_ISL_436072	EPI_ISL_418199	EPI_ISL_421392
EPI_ISL_427585	EPI_ISL_434282	EPI_ISL_436073	EPI_ISL_420090	EPI_ISL_421393
EPI_ISL_427586	EPI_ISL_434283	EPI_ISL_436074	EPI_ISL_420093	EPI_ISL_421394
EPI_ISL_427587	EPI_ISL_434284	EPI_ISL_436075	EPI_ISL_420094	EPI_ISL_421395
EPI_ISL_427588	EPI_ISL_434285	EPI_ISL_436076	EPI_ISL_421348	EPI_ISL_421396
EPI_ISL_427589	EPI_ISL_434286	EPI_ISL_436077	EPI_ISL_421349	EPI_ISL_421397
EPI_ISL_427590	EPI_ISL_434287	EPI_ISL_436078	EPI_ISL_421350	EPI_ISL_421398
EPI_ISL_427591	EPI_ISL_434288	EPI_ISL_436079	EPI_ISL_421351	EPI_ISL_421399
EPI_ISL_427592	EPI_ISL_434289	EPI_ISL_436080	EPI_ISL_421352	EPI_ISL_426617
EPI_ISL_427593	EPI_ISL_434290	EPI_ISL_436081	EPI_ISL_421353	EPI_ISL_426618
EPI_ISL_427594	EPI_ISL_434291	EPI_ISL_436082	EPI_ISL_421354	EPI_ISL_426619
EPI_ISL_427595	EPI_ISL_434292	EPI_ISL_438200	EPI_ISL_421355	EPI_ISL_426620
EPI_ISL_427596	EPI_ISL_434293	EPI_ISL_438201	EPI_ISL_421356	EPI_ISL_426621
EPI_ISL_427597	EPI_ISL_434294	EPI_ISL_438202	EPI_ISL_421357	EPI_ISL_426622
EPI_ISL_427598	EPI_ISL_434295	EPI_ISL_438203	EPI_ISL_421358	EPI_ISL_426623
EPI_ISL_427599	EPI_ISL_434296	EPI_ISL_438204	EPI_ISL_421359	EPI_ISL_426624
EPI_ISL_430323	EPI_ISL_434297	EPI_ISL_438205	EPI_ISL_421360	EPI_ISL_426625
EPI_ISL_430324	EPI_ISL_434298	EPI_ISL_438206	EPI_ISL_421361	EPI_ISL_426626
EPI_ISL_430325	EPI_ISL_434299	EPI_ISL_438207	EPI_ISL_421362	EPI_ISL_427528
EPI_ISL_430326	EPI_ISL_436040	EPI_ISL_438208	EPI_ISL_421363	EPI_ISL_427529
EPI_ISL_430327	EPI_ISL_436041	EPI_ISL_438209	EPI_ISL_421364	EPI_ISL_427530
EPI_ISL_430328	EPI_ISL_436042	EPI_ISL_438218	EPI_ISL_421365	EPI_ISL_427531
EPI_ISL_430329	EPI_ISL_436043	EPI_ISL_438219	EPI_ISL_421366	EPI_ISL_427532
EPI_ISL_430330	EPI_ISL_436047	EPI_ISL_438220	EPI_ISL_421367	EPI_ISL_427533
EPI_ISL_430331	EPI_ISL_436048	EPI_ISL_438221	EPI_ISL_421368	EPI_ISL_427534
EPI_ISL_430332	EPI_ISL_436049	EPI_ISL_414616	EPI_ISL_421369	EPI_ISL_427535

© 2020 Zhang W et al. *JAMA Network Open.*

EPI_ISL_427536	EPI_ISL_434219	EPI_ISL_435510	EPI_ISL_443186	EPI_ISL_418775
EPI_ISL_427537	EPI_ISL_434220	EPI_ISL_435511	EPI_ISL_444634	EPI_ISL_418776
EPI_ISL_427538	EPI_ISL_434221	EPI_ISL_435512	EPI_ISL_444635	EPI_ISL_418777
EPI_ISL_427539	EPI_ISL_434222	EPI_ISL_435513	EPI_ISL_444636	EPI_ISL_418778
EPI_ISL_427540	EPI_ISL_434223	EPI_ISL_435514	EPI_ISL_444640	EPI_ISL_418779
EPI_ISL_427541	EPI_ISL_434224	EPI_ISL_435515	EPI_ISL_444641	EPI_ISL_419696
EPI_ISL_427542	EPI_ISL_434225	EPI_ISL_435516	EPI_ISL_444642	EPI_ISL_419697
EPI_ISL_427543	EPI_ISL_434226	EPI_ISL_435517	EPI_ISL_444643	EPI_ISL_419698
EPI_ISL_427544	EPI_ISL_434227	EPI_ISL_435518	EPI_ISL_450089	EPI_ISL_419699
EPI_ISL_427545	EPI_ISL_434228	EPI_ISL_435520	EPI_ISL_450090	EPI_ISL_421577
EPI_ISL_427546	EPI_ISL_434229	EPI_ISL_435521	EPI_ISL_450091	EPI_ISL_421578
EPI_ISL_427547	EPI_ISL_434230	EPI_ISL_435522	EPI_ISL_450096	EPI_ISL_421579
EPI_ISL_427548	EPI_ISL_434231	EPI_ISL_435523	EPI_ISL_450097	EPI_ISL_421580
EPI_ISL_427549	EPI_ISL_434232	EPI_ISL_435524	EPI_ISL_450098	EPI_ISL_421581
EPI_ISL_427560	EPI_ISL_434233	EPI_ISL_435525	EPI_ISL_450099	EPI_ISL_421582
EPI_ISL_427561	EPI_ISL_434234	EPI_ISL_435526	EPI_ISL_407214	EPI_ISL_421583
EPI_ISL_427562	EPI_ISL_434235	EPI_ISL_435527	EPI_ISL_407215	EPI_ISL_421586
EPI_ISL_427563	EPI_ISL_434236	EPI_ISL_435528	EPI_ISL_417065	EPI_ISL_421587
EPI_ISL_427564	EPI_ISL_434237	EPI_ISL_435529	EPI_ISL_417066	EPI_ISL_424180
EPI_ISL_427565	EPI_ISL_434238	EPI_ISL_435530	EPI_ISL_417067	EPI_ISL_424182
EPI_ISL_427566	EPI_ISL_434239	EPI_ISL_435531	EPI_ISL_417068	EPI_ISL_424183
EPI_ISL_427567	EPI_ISL_434240	EPI_ISL_435532	EPI_ISL_417069	EPI_ISL_424184
EPI_ISL_427568	EPI_ISL_434241	EPI_ISL_435533	EPI_ISL_417070	EPI_ISL_424186
EPI_ISL_427569	EPI_ISL_434242	EPI_ISL_435534	EPI_ISL_417071	EPI_ISL_424187
EPI_ISL_428800	EPI_ISL_434243	EPI_ISL_435535	EPI_ISL_417072	EPI_ISL_424188
EPI_ISL_428801	EPI_ISL_434244	EPI_ISL_435536	EPI_ISL_417073	EPI_ISL_424190
EPI_ISL_428802	EPI_ISL_434245	EPI_ISL_435537	EPI_ISL_417074	EPI_ISL_424191
EPI_ISL_428803	EPI_ISL_434246	EPI_ISL_435538	EPI_ISL_417075	EPI_ISL_424192
EPI_ISL_428804	EPI_ISL_434247	EPI_ISL_435539	EPI_ISL_417076	EPI_ISL_424193
EPI_ISL_428805	EPI_ISL_434248	EPI_ISL_435540	EPI_ISL_417077	EPI_ISL_424194
EPI_ISL_434200	EPI_ISL_434249	EPI_ISL_435541	EPI_ISL_417078	EPI_ISL_424195
EPI_ISL_434201	EPI_ISL_434250	EPI_ISL_435542	EPI_ISL_417079	EPI_ISL_424196
EPI_ISL_434202	EPI_ISL_434251	EPI_ISL_435543	EPI_ISL_417080	EPI_ISL_424197
EPI_ISL_434204	EPI_ISL_434252	EPI_ISL_435544	EPI_ISL_417081	EPI_ISL_424198
EPI_ISL_434205	EPI_ISL_434253	EPI_ISL_435545	EPI_ISL_417082	EPI_ISL_424199
EPI_ISL_434206	EPI_ISL_434254	EPI_ISL_435546	EPI_ISL_417083	EPI_ISL_427209
EPI_ISL_434207	EPI_ISL_434255	EPI_ISL_435547	EPI_ISL_417084	EPI_ISL_427210
EPI_ISL_434208	EPI_ISL_434256	EPI_ISL_435548	EPI_ISL_417085	EPI_ISL_427211
EPI_ISL_434209	EPI_ISL_434257	EPI_ISL_435549	EPI_ISL_417086	EPI_ISL_427212
EPI_ISL_434210	EPI_ISL_434258	EPI_ISL_438210	EPI_ISL_417087	EPI_ISL_427213
EPI_ISL_434211	EPI_ISL_434259	EPI_ISL_438211	EPI_ISL_417088	EPI_ISL_427214
EPI_ISL_434212	EPI_ISL_435503	EPI_ISL_438212	EPI_ISL_417089	EPI_ISL_427215
EPI_ISL_434213	EPI_ISL_435504	EPI_ISL_438213	EPI_ISL_417448	EPI_ISL_427216
EPI_ISL_434214	EPI_ISL_435505	EPI_ISL_438214	EPI_ISL_417449	EPI_ISL_427217
EPI_ISL_434215	EPI_ISL_435506	EPI_ISL_438215	EPI_ISL_418771	EPI_ISL_427218
EPI_ISL_434216	EPI_ISL_435507	EPI_ISL_438216	EPI_ISL_418772	EPI_ISL_427219
EPI_ISL_434217	EPI_ISL_435508	EPI_ISL_438217	EPI_ISL_418773	EPI_ISL_427220
EPI_ISL_434218	EPI_ISL_435509	EPI_ISL_443184	EPI_ISL_418774	EPI_ISL_427221

© 2020 Zhang W et al. *JAMA Network Open.*

EPI_ISL_427222	EPI_ISL_430891	EPI_ISL_444665	EPI_ISL_450061	EPI_ISL_417451
EPI_ISL_427230	EPI_ISL_430892	EPI_ISL_444666	EPI_ISL_450062	EPI_ISL_417453
EPI_ISL_427231	EPI_ISL_430893	EPI_ISL_444667	EPI_ISL_450063	EPI_ISL_417454
EPI_ISL_427232	EPI_ISL_435721	EPI_ISL_444668	EPI_ISL_450064	EPI_ISL_417455
EPI_ISL_427233	EPI_ISL_435722	EPI_ISL_444669	EPI_ISL_450065	EPI_ISL_417456
EPI_ISL_427234	EPI_ISL_444613	EPI_ISL_444671	EPI_ISL_450066	EPI_ISL_418780
EPI_ISL_427235	EPI_ISL_444614	EPI_ISL_444673	EPI_ISL_450067	EPI_ISL_418781
EPI_ISL_427236	EPI_ISL_444615	EPI_ISL_444675	EPI_ISL_450068	EPI_ISL_418782
EPI_ISL_427237	EPI_ISL_444616	EPI_ISL_444677	EPI_ISL_450069	EPI_ISL_418783
EPI_ISL_427238	EPI_ISL_444617	EPI_ISL_444678	EPI_ISL_450070	EPI_ISL_418784
EPI_ISL_427239	EPI_ISL_444618	EPI_ISL_444679	EPI_ISL_450071	EPI_ISL_418785
EPI_ISL_427240	EPI_ISL_444619	EPI_ISL_444680	EPI_ISL_450072	EPI_ISL_418786
EPI_ISL_427241	EPI_ISL_444620	EPI_ISL_444681	EPI_ISL_450074	EPI_ISL_418787
EPI_ISL_427242	EPI_ISL_444621	EPI_ISL_444682	EPI_ISL_450075	EPI_ISL_418788
EPI_ISL_427243	EPI_ISL_444622	EPI_ISL_444683	EPI_ISL_450076	EPI_ISL_418789
EPI_ISL_427244	EPI_ISL_444623	EPI_ISL_444684	EPI_ISL_450077	EPI_ISL_418790
EPI_ISL_427245	EPI_ISL_444624	EPI_ISL_444685	EPI_ISL_450078	EPI_ISL_418791
EPI_ISL_427246	EPI_ISL_444625	EPI_ISL_444686	EPI_ISL_450079	EPI_ISL_424166
EPI_ISL_427247	EPI_ISL_444626	EPI_ISL_444687	EPI_ISL_450080	EPI_ISL_424167
EPI_ISL_427248	EPI_ISL_444627	EPI_ISL_444688	EPI_ISL_450081	EPI_ISL_424168
EPI_ISL_427249	EPI_ISL_444628	EPI_ISL_444690	EPI_ISL_450082	EPI_ISL_424169
EPI_ISL_430400	EPI_ISL_444629	EPI_ISL_444691	EPI_ISL_450083	EPI_ISL_424170
EPI_ISL_430402	EPI_ISL_444630	EPI_ISL_444692	EPI_ISL_450084	EPI_ISL_424171
EPI_ISL_430403	EPI_ISL_444631	EPI_ISL_444693	EPI_ISL_450085	EPI_ISL_424172
EPI_ISL_430404	EPI_ISL_444632	EPI_ISL_444694	EPI_ISL_450086	EPI_ISL_424175
EPI_ISL_430406	EPI_ISL_444633	EPI_ISL_444695	EPI_ISL_450087	EPI_ISL_424176
EPI_ISL_430407	EPI_ISL_444637	EPI_ISL_444696	EPI_ISL_450088	EPI_ISL_424177
EPI_ISL_430408	EPI_ISL_444638	EPI_ISL_444697	EPI_ISL_450092	EPI_ISL_424178
EPI_ISL_430409	EPI_ISL_444639	EPI_ISL_444698	EPI_ISL_450093	EPI_ISL_424179
EPI_ISL_430867	EPI_ISL_444644	EPI_ISL_449900	EPI_ISL_450094	EPI_ISL_426300
EPI_ISL_430868	EPI_ISL_444645	EPI_ISL_449901	EPI_ISL_450095	EPI_ISL_426301
EPI_ISL_430869	EPI_ISL_444646	EPI_ISL_450000	EPI_ISL_413025	EPI_ISL_426302
EPI_ISL_430870	EPI_ISL_444647	EPI_ISL_450001	EPI_ISL_413455	EPI_ISL_426303
EPI_ISL_430871	EPI_ISL_444648	EPI_ISL_450002	EPI_ISL_413456	EPI_ISL_426304
EPI_ISL_430872	EPI_ISL_444650	EPI_ISL_450003	EPI_ISL_413457	EPI_ISL_426305
EPI_ISL_430873	EPI_ISL_444651	EPI_ISL_450004	EPI_ISL_413458	EPI_ISL_426306
EPI_ISL_430874	EPI_ISL_444652	EPI_ISL_450005	EPI_ISL_413486	EPI_ISL_426307
EPI_ISL_430875	EPI_ISL_444653	EPI_ISL_450006	EPI_ISL_413487	EPI_ISL_426308
EPI_ISL_430876	EPI_ISL_444654	EPI_ISL_450007	EPI_ISL_414363	EPI_ISL_426309
EPI_ISL_430877	EPI_ISL_444656	EPI_ISL_450008	EPI_ISL_414364	EPI_ISL_426310
EPI_ISL_430878	EPI_ISL_444657	EPI_ISL_450009	EPI_ISL_414365	EPI_ISL_426311
EPI_ISL_430880	EPI_ISL_444658	EPI_ISL_450010	EPI_ISL_414366	EPI_ISL_426312
EPI_ISL_430883	EPI_ISL_444659	EPI_ISL_450011	EPI_ISL_414367	EPI_ISL_426313
EPI_ISL_430884	EPI_ISL_444660	EPI_ISL_450056	EPI_ISL_414368	EPI_ISL_426314
EPI_ISL_430885	EPI_ISL_444661	EPI_ISL_450057	EPI_ISL_414369	EPI_ISL_426315
EPI_ISL_430887	EPI_ISL_444662	EPI_ISL_450058	EPI_ISL_415626	EPI_ISL_426316
EPI_ISL_430888	EPI_ISL_444663	EPI_ISL_450059	EPI_ISL_415627	EPI_ISL_426317
EPI_ISL_430889	EPI_ISL_444664	EPI_ISL_450060	EPI_ISL_417450	EPI_ISL_426318

© 2020 Zhang W et al. *JAMA Network Open.*

EPI_ISL_426319	EPI_ISL_427607	EPI_ISL_430431	EPI_ISL_449896	EPI_ISL_415602
EPI_ISL_426320	EPI_ISL_427608	EPI_ISL_430432	EPI_ISL_449897	EPI_ISL_415603
EPI_ISL_426321	EPI_ISL_427609	EPI_ISL_430433	EPI_ISL_449898	EPI_ISL_415604
EPI_ISL_426322	EPI_ISL_427610	EPI_ISL_430434	EPI_ISL_449899	EPI_ISL_415605
EPI_ISL_426323	EPI_ISL_427611	EPI_ISL_430894	EPI_ISL_450012	EPI_ISL_415606
EPI_ISL_426324	EPI_ISL_427612	EPI_ISL_430895	EPI_ISL_450013	EPI_ISL_415607
EPI_ISL_426326	EPI_ISL_427613	EPI_ISL_430896	EPI_ISL_450014	EPI_ISL_415608
EPI_ISL_426327	EPI_ISL_427614	EPI_ISL_430898	EPI_ISL_450015	EPI_ISL_415609
EPI_ISL_426328	EPI_ISL_427615	EPI_ISL_430899	EPI_ISL_450016	EPI_ISL_415610
EPI_ISL_427200	EPI_ISL_427616	EPI_ISL_434315	EPI_ISL_450017	EPI_ISL_415611
EPI_ISL_427201	EPI_ISL_427617	EPI_ISL_434316	EPI_ISL_450018	EPI_ISL_415612
EPI_ISL_427202	EPI_ISL_427618	EPI_ISL_434317	EPI_ISL_450019	EPI_ISL_415613
EPI_ISL_427203	EPI_ISL_427627	EPI_ISL_434318	EPI_ISL_450020	EPI_ISL_415614
EPI_ISL_427204	EPI_ISL_427628	EPI_ISL_434319	EPI_ISL_450021	EPI_ISL_415615
EPI_ISL_427205	EPI_ISL_427629	EPI_ISL_434322	EPI_ISL_450022	EPI_ISL_415616
EPI_ISL_427206	EPI_ISL_427630	EPI_ISL_434323	EPI_ISL_450023	EPI_ISL_415617
EPI_ISL_427207	EPI_ISL_427631	EPI_ISL_434324	EPI_ISL_450024	EPI_ISL_415618
EPI_ISL_427208	EPI_ISL_427632	EPI_ISL_434325	EPI_ISL_450025	EPI_ISL_415619
EPI_ISL_427223	EPI_ISL_427633	EPI_ISL_437824	EPI_ISL_450026	EPI_ISL_415620
EPI_ISL_427224	EPI_ISL_427634	EPI_ISL_437827	EPI_ISL_450027	EPI_ISL_415621
EPI_ISL_427225	EPI_ISL_427635	EPI_ISL_437829	EPI_ISL_450028	EPI_ISL_415622
EPI_ISL_427226	EPI_ISL_427636	EPI_ISL_449869	EPI_ISL_450029	EPI_ISL_415623
EPI_ISL_427227	EPI_ISL_427637	EPI_ISL_449870	EPI_ISL_450030	EPI_ISL_415624
EPI_ISL_427228	EPI_ISL_427638	EPI_ISL_449871	EPI_ISL_450031	EPI_ISL_415625
EPI_ISL_427229	EPI_ISL_427639	EPI_ISL_449872	EPI_ISL_450032	EPI_ISL_427600
EPI_ISL_427250	EPI_ISL_427640	EPI_ISL_449873	EPI_ISL_450033	EPI_ISL_427601
EPI_ISL_427251	EPI_ISL_427641	EPI_ISL_449874	EPI_ISL_450034	EPI_ISL_427602
EPI_ISL_427252	EPI_ISL_427642	EPI_ISL_449875	EPI_ISL_450035	EPI_ISL_427603
EPI_ISL_427253	EPI_ISL_430410	EPI_ISL_449876	EPI_ISL_450036	EPI_ISL_427604
EPI_ISL_427254	EPI_ISL_430411	EPI_ISL_449877	EPI_ISL_450037	EPI_ISL_434300
EPI_ISL_427255	EPI_ISL_430412	EPI_ISL_449878	EPI_ISL_450038	EPI_ISL_434301
EPI_ISL_427256	EPI_ISL_430413	EPI_ISL_449879	EPI_ISL_450039	EPI_ISL_434302
EPI_ISL_427257	EPI_ISL_430414	EPI_ISL_449880	EPI_ISL_450040	EPI_ISL_434303
EPI_ISL_427258	EPI_ISL_430415	EPI_ISL_449881	EPI_ISL_450041	EPI_ISL_434304
EPI_ISL_427259	EPI_ISL_430416	EPI_ISL_449882	EPI_ISL_450042	EPI_ISL_434305
EPI_ISL_427260	EPI_ISL_430417	EPI_ISL_449883	EPI_ISL_450043	EPI_ISL_434306
EPI_ISL_427261	EPI_ISL_430418	EPI_ISL_449884	EPI_ISL_450044	EPI_ISL_434307
EPI_ISL_427262	EPI_ISL_430419	EPI_ISL_449885	EPI_ISL_450045	EPI_ISL_434308
EPI_ISL_427263	EPI_ISL_430420	EPI_ISL_449886	EPI_ISL_450046	EPI_ISL_434309
EPI_ISL_427264	EPI_ISL_430421	EPI_ISL_449887	EPI_ISL_450047	EPI_ISL_434310
EPI_ISL_427265	EPI_ISL_430422	EPI_ISL_449888	EPI_ISL_450048	EPI_ISL_434311
EPI_ISL_427266	EPI_ISL_430423	EPI_ISL_449889	EPI_ISL_450050	EPI_ISL_434312
EPI_ISL_427267	EPI_ISL_430424	EPI_ISL_449890	EPI_ISL_450051	EPI_ISL_434313
EPI_ISL_427268	EPI_ISL_430425	EPI_ISL_449891	EPI_ISL_450052	EPI_ISL_434314
EPI_ISL_427269	EPI_ISL_430426	EPI_ISL_449892	EPI_ISL_450053	EPI_ISL_434320
EPI_ISL_427270	EPI_ISL_430427	EPI_ISL_449893	EPI_ISL_450054	EPI_ISL_434321
EPI_ISL_427605	EPI_ISL_430429	EPI_ISL_449894	EPI_ISL_450055	EPI_ISL_434326
EPI_ISL_427606	EPI_ISL_430430	EPI_ISL_449895	EPI_ISL_415601	EPI_ISL_434327

© 2020 Zhang W et al. *JAMA Network Open.*

EPI_ISL_434328	EPI_ISL_437850	EPI_ISL_406531	EPI_ISL_413901	EPI_ISL_416358
EPI_ISL_434329	EPI_ISL_437851	EPI_ISL_406533	EPI_ISL_413902	EPI_ISL_416359
EPI_ISL_434330	EPI_ISL_437853	EPI_ISL_406534	EPI_ISL_414663	EPI_ISL_416360
EPI_ISL_434331	EPI_ISL_437854	EPI_ISL_406535	EPI_ISL_414690	EPI_ISL_416361
EPI_ISL_434332	EPI_ISL_437855	EPI_ISL_406536	EPI_ISL_414691	EPI_ISL_416362
EPI_ISL_434333	EPI_ISL_437856	EPI_ISL_406538	EPI_ISL_416042	EPI_ISL_416363
EPI_ISL_434334	EPI_ISL_437857	EPI_ISL_406592	EPI_ISL_416044	EPI_ISL_416364
EPI_ISL_434335	EPI_ISL_437858	EPI_ISL_406593	EPI_ISL_416046	EPI_ISL_416365
EPI_ISL_434336	EPI_ISL_437859	EPI_ISL_406594	EPI_ISL_416047	EPI_ISL_416366
EPI_ISL_434337	EPI_ISL_437860	EPI_ISL_406595	EPI_ISL_416319	EPI_ISL_416367
EPI_ISL_434338	EPI_ISL_437861	EPI_ISL_406798	EPI_ISL_416320	EPI_ISL_416368
EPI_ISL_434339	EPI_ISL_437862	EPI_ISL_406799	EPI_ISL_416321	EPI_ISL_416369
EPI_ISL_434340	EPI_ISL_437863	EPI_ISL_406800	EPI_ISL_416322	EPI_ISL_416370
EPI_ISL_434341	EPI_ISL_437864	EPI_ISL_406801	EPI_ISL_416323	EPI_ISL_416371
EPI_ISL_434342	EPI_ISL_449839	EPI_ISL_407313	EPI_ISL_416324	EPI_ISL_416372
EPI_ISL_434343	EPI_ISL_449840	EPI_ISL_408478	EPI_ISL_416325	EPI_ISL_416373
EPI_ISL_434344	EPI_ISL_449841	EPI_ISL_408479	EPI_ISL_416326	EPI_ISL_416374
EPI_ISL_434345	EPI_ISL_449842	EPI_ISL_408480	EPI_ISL_416327	EPI_ISL_416375
EPI_ISL_434346	EPI_ISL_449843	EPI_ISL_408481	EPI_ISL_416328	EPI_ISL_416376
EPI_ISL_437804	EPI_ISL_449844	EPI_ISL_408482	EPI_ISL_416329	EPI_ISL_416377
EPI_ISL_437805	EPI_ISL_449845	EPI_ISL_408483	EPI_ISL_416330	EPI_ISL_416378
EPI_ISL_437806	EPI_ISL_449846	EPI_ISL_408484	EPI_ISL_416331	EPI_ISL_416379
EPI_ISL_437807	EPI_ISL_449847	EPI_ISL_408485	EPI_ISL_416332	EPI_ISL_416380
EPI_ISL_437808	EPI_ISL_449848	EPI_ISL_408486	EPI_ISL_416333	EPI_ISL_416381
EPI_ISL_437809	EPI_ISL_449849	EPI_ISL_408487	EPI_ISL_416334	EPI_ISL_416382
EPI_ISL_437810	EPI_ISL_449850	EPI_ISL_408488	EPI_ISL_416335	EPI_ISL_416383
EPI_ISL_437811	EPI_ISL_449851	EPI_ISL_408978	EPI_ISL_416336	EPI_ISL_416384
EPI_ISL_437812	EPI_ISL_449852	EPI_ISL_411950	EPI_ISL_416337	EPI_ISL_416385
EPI_ISL_437813	EPI_ISL_449853	EPI_ISL_411952	EPI_ISL_416338	EPI_ISL_416386
EPI_ISL_437814	EPI_ISL_449854	EPI_ISL_411953	EPI_ISL_416339	EPI_ISL_416387
EPI_ISL_437815	EPI_ISL_449855	EPI_ISL_411957	EPI_ISL_416340	EPI_ISL_416388
EPI_ISL_437816	EPI_ISL_449856	EPI_ISL_412900	EPI_ISL_416341	EPI_ISL_416389
EPI_ISL_437817	EPI_ISL_449857	EPI_ISL_412966	EPI_ISL_416342	EPI_ISL_416390
EPI_ISL_437820	EPI_ISL_449858	EPI_ISL_412967	EPI_ISL_416343	EPI_ISL_416391
EPI_ISL_437821	EPI_ISL_449859	EPI_ISL_412978	EPI_ISL_416344	EPI_ISL_416392
EPI_ISL_437822	EPI_ISL_449860	EPI_ISL_412979	EPI_ISL_416345	EPI_ISL_416393
EPI_ISL_437823	EPI_ISL_449861	EPI_ISL_412980	EPI_ISL_416346	EPI_ISL_416394
EPI_ISL_437839	EPI_ISL_449862	EPI_ISL_412981	EPI_ISL_416347	EPI_ISL_416395
EPI_ISL_437840	EPI_ISL_449863	EPI_ISL_412982	EPI_ISL_416348	EPI_ISL_416396
EPI_ISL_437841	EPI_ISL_449864	EPI_ISL_412983	EPI_ISL_416349	EPI_ISL_416397
EPI_ISL_437842	EPI_ISL_449865	EPI_ISL_413691	EPI_ISL_416350	EPI_ISL_416398
EPI_ISL_437843	EPI_ISL_449866	EPI_ISL_413692	EPI_ISL_416351	EPI_ISL_416399
EPI_ISL_437844	EPI_ISL_449867	EPI_ISL_413693	EPI_ISL_416352	EPI_ISL_416473
EPI_ISL_437845	EPI_ISL_449868	EPI_ISL_413694	EPI_ISL_416353	EPI_ISL_416474
EPI_ISL_437846	EPI_ISL_404227	EPI_ISL_413697	EPI_ISL_416354	EPI_ISL_417420
EPI_ISL_437847	EPI_ISL_404228	EPI_ISL_413711	EPI_ISL_416355	EPI_ISL_418441
EPI_ISL_437848	EPI_ISL_405839	EPI_ISL_413809	EPI_ISL_416356	EPI_ISL_418442
EPI_ISL_437849	EPI_ISL_406030	EPI_ISL_413900	EPI_ISL_416357	EPI_ISL_418502

© 2020 Zhang W et al. *JAMA Network Open.*

EPI_ISL_418503	EPI_ISL_428464	EPI_ISL_402123	EPI_ISL_413856	EPI_ISL_414689
EPI_ISL_418504	EPI_ISL_428465	EPI_ISL_402124	EPI_ISL_413857	EPI_ISL_414692
EPI_ISL_418506	EPI_ISL_428466	EPI_ISL_402125	EPI_ISL_413858	EPI_ISL_414934
EPI_ISL_418508	EPI_ISL_428467	EPI_ISL_402127	EPI_ISL_413859	EPI_ISL_414935
EPI_ISL_418509	EPI_ISL_428468	EPI_ISL_402128	EPI_ISL_413860	EPI_ISL_414936
EPI_ISL_418510	EPI_ISL_428469	EPI_ISL_402129	EPI_ISL_413861	EPI_ISL_414937
EPI_ISL_418511	EPI_ISL_428470	EPI_ISL_402130	EPI_ISL_413862	EPI_ISL_414938
EPI_ISL_418512	EPI_ISL_428471	EPI_ISL_402132	EPI_ISL_413863	EPI_ISL_414939
EPI_ISL_418513	EPI_ISL_428472	EPI_ISL_403928	EPI_ISL_413864	EPI_ISL_414940
EPI_ISL_418514	EPI_ISL_428473	EPI_ISL_403929	EPI_ISL_413865	EPI_ISL_414941
EPI_ISL_418515	EPI_ISL_428474	EPI_ISL_403930	EPI_ISL_413866	EPI_ISL_415709
EPI_ISL_418990	EPI_ISL_428475	EPI_ISL_403931	EPI_ISL_413867	EPI_ISL_415711
EPI_ISL_418991	EPI_ISL_428476	EPI_ISL_403932	EPI_ISL_413868	EPI_ISL_416316
EPI_ISL_421221	EPI_ISL_428477	EPI_ISL_403933	EPI_ISL_413869	EPI_ISL_416317
EPI_ISL_421222	EPI_ISL_428478	EPI_ISL_403934	EPI_ISL_413870	EPI_ISL_416318
EPI_ISL_421223	EPI_ISL_429100	EPI_ISL_403935	EPI_ISL_413871	EPI_ISL_416400
EPI_ISL_421224	EPI_ISL_429101	EPI_ISL_403936	EPI_ISL_413872	EPI_ISL_416401
EPI_ISL_422425	EPI_ISL_429102	EPI_ISL_403937	EPI_ISL_413873	EPI_ISL_416402
EPI_ISL_424352	EPI_ISL_429103	EPI_ISL_406716	EPI_ISL_413874	EPI_ISL_416403
EPI_ISL_424355	EPI_ISL_429104	EPI_ISL_406717	EPI_ISL_413875	EPI_ISL_416404
EPI_ISL_424356	EPI_ISL_429105	EPI_ISL_406970	EPI_ISL_413876	EPI_ISL_416405
EPI_ISL_424357	EPI_ISL_429239	EPI_ISL_411060	EPI_ISL_413877	EPI_ISL_416406
EPI_ISL_424358	EPI_ISL_430730	EPI_ISL_411066	EPI_ISL_413878	EPI_ISL_416407
EPI_ISL_424359	EPI_ISL_430731	EPI_ISL_412026	EPI_ISL_413879	EPI_ISL_416408
EPI_ISL_428440	EPI_ISL_430733	EPI_ISL_412459	EPI_ISL_413880	EPI_ISL_416409
EPI_ISL_428441	EPI_ISL_430734	EPI_ISL_412898	EPI_ISL_413881	EPI_ISL_416425
EPI_ISL_428442	EPI_ISL_430735	EPI_ISL_412899	EPI_ISL_413882	EPI_ISL_421225
EPI_ISL_428443	EPI_ISL_430736	EPI_ISL_413485	EPI_ISL_413883	EPI_ISL_421226
EPI_ISL_428444	EPI_ISL_430737	EPI_ISL_413518	EPI_ISL_413884	EPI_ISL_421227
EPI_ISL_428445	EPI_ISL_430738	EPI_ISL_413519	EPI_ISL_413885	EPI_ISL_421228
EPI_ISL_428446	EPI_ISL_430739	EPI_ISL_413520	EPI_ISL_413886	EPI_ISL_421229
EPI_ISL_428447	EPI_ISL_430740	EPI_ISL_413521	EPI_ISL_413887	EPI_ISL_421230
EPI_ISL_428448	EPI_ISL_430741	EPI_ISL_413729	EPI_ISL_413888	EPI_ISL_421231
EPI_ISL_428449	EPI_ISL_430742	EPI_ISL_413746	EPI_ISL_413889	EPI_ISL_421232
EPI_ISL_428450	EPI_ISL_431118	EPI_ISL_413748	EPI_ISL_413890	EPI_ISL_421233
EPI_ISL_428451	EPI_ISL_431240	EPI_ISL_413749	EPI_ISL_413891	EPI_ISL_421234
EPI_ISL_428452	EPI_ISL_431779	EPI_ISL_413750	EPI_ISL_413892	EPI_ISL_421235
EPI_ISL_428453	EPI_ISL_431780	EPI_ISL_413751	EPI_ISL_413893	EPI_ISL_421236
EPI_ISL_428454	EPI_ISL_431781	EPI_ISL_413752	EPI_ISL_413894	EPI_ISL_421237
EPI_ISL_428455	EPI_ISL_431782	EPI_ISL_413753	EPI_ISL_413895	EPI_ISL_421238
EPI_ISL_428456	EPI_ISL_431783	EPI_ISL_413761	EPI_ISL_413896	EPI_ISL_421239
EPI_ISL_428457	EPI_ISL_431784	EPI_ISL_413791	EPI_ISL_413897	EPI_ISL_421240
EPI_ISL_428458	EPI_ISL_431785	EPI_ISL_413850	EPI_ISL_413898	EPI_ISL_421241
EPI_ISL_428459	EPI_ISL_434534	EPI_ISL_413851	EPI_ISL_413899	EPI_ISL_421242
EPI_ISL_428460	EPI_ISL_444969	EPI_ISL_413852	EPI_ISL_414510	EPI_ISL_421243
EPI_ISL_428461	EPI_ISL_402119	EPI_ISL_413853	EPI_ISL_414686	EPI_ISL_421244
EPI_ISL_428462	EPI_ISL_402120	EPI_ISL_413854	EPI_ISL_414687	EPI_ISL_421245
EPI_ISL_428463	EPI_ISL_402121	EPI_ISL_413855	EPI_ISL_414688	EPI_ISL_421246

© 2020 Zhang W et al. *JAMA Network Open.*

EPI_ISL_421247	EPI_ISL_421259	EPI_ISL_429080	EPI_ISL_429092	EPI_ISL_429855
EPI_ISL_421248	EPI_ISL_421260	EPI_ISL_429081	EPI_ISL_429093	EPI_ISL_430722
EPI_ISL_421249	EPI_ISL_421261	EPI_ISL_429082	EPI_ISL_429094	EPI_ISL_430724
EPI_ISL_421250	EPI_ISL_421262	EPI_ISL_429083	EPI_ISL_429095	EPI_ISL_430725
EPI_ISL_421251	EPI_ISL_424360	EPI_ISL_429084	EPI_ISL_429096	EPI_ISL_430728
EPI_ISL_421252	EPI_ISL_429074	EPI_ISL_429085	EPI_ISL_429097	EPI_ISL_430729
EPI_ISL_421253	EPI_ISL_429075	EPI_ISL_429086	EPI_ISL_429098	EPI_ISL_431180
EPI_ISL_421254	EPI_ISL_429076	EPI_ISL_429088	EPI_ISL_429099	EPI_ISL_431292
EPI_ISL_421256	EPI_ISL_429077	EPI_ISL_429089	EPI_ISL_429852	EPI_ISL_444273
EPI_ISL_421257	EPI_ISL_429078	EPI_ISL_429090	EPI_ISL_429853	
EPI_ISL_421258	EPI_ISL_429079	EPI_ISL_429091	EPI_ISL_429854	

© 2020 Zhang W et al. *JAMA Network Open.*
